# Inter- and intra-household perceived relative inequality among disabled and non-disabled people in Liberia

**DOI:** 10.1371/journal.pone.0217873

**Published:** 2019-07-17

**Authors:** Mark T. Carew, Tim Colbourn, Ellie Cole, Richard Ngafuan, Nora Groce, Maria Kett

**Affiliations:** 1 Leonard Cheshire Research Centre, University College London, London, United Kingdom; 2 Institute for Global Health, University College London, London, United Kingdom; 3 University of Liberia, Monrovia, Liberia; Università degli Studi di Perugia, ITALY

## Abstract

Evidence suggests that people with disabilities are the most marginalised and vulnerable group within any population. However, little is known about the extent of inequality between people with and without disabilities in contexts where the majority of persons experience extreme poverty and hardship. This includes in Liberia, where very little is understood about the lives of disabled people in general. This study uses a multidimensional wellbeing framework to understand perceived relative inequality associated with disability by assessing several facets of wellbeing across and within households containing disabled members (*N* = 485) or households with no disabled members (*N* = 538) in Liberian communities (Total individuals surveyed, *N* = 2020). Statistical comparisons (adjusted for age, sex, education and wealth differences and clustered at the household, village and county level) reveal that disabled Liberians are managing similarly to non-disabled Liberians in terms of income and education, but experience many perceived relative inequalities including in life satisfaction, transport access, political participation and social inclusion. Our results further suggest that disability may lead to perceived relative inequality at the household level in terms of trust held in neighbours. However, they also show that being the head of a household may protect against perceived relative inequality in certain dimensions (e.g. healthcare and transport access, political participation) irrespective of disability status. Results are discussed in terms of practical implications for development efforts in Liberia and for disabled people in other low- and middle-income settings.

## Introduction

This paper sets out to explore the issue of perceived relative inequality in Liberia using a multidimensional well-being framework, with particular attention to how people with disabilities compare to their non-disabled peers. In recent years, evidence has accumulated to show that people with disabilities are the most marginalised and vulnerable group within any population [1, 2] and may be the world’s largest minority [3]. It is perhaps small wonder then that international development efforts in low- and middle-income countries–where 80% of the global population of disabled people are estimated to reside [4]- have begun to explicitly target, as well as grown steadily more inclusive of, people with disabilities. This work is underpinned by several international human rights conventions and declarations that both protect the rights of persons with disabilities [5]; as well as set global goals that constitute a call to action for international actors regarding what is needed so that *all* persons, including those with disabilities, can enjoy prosperity and wellbeing (e.g., the Sustainable Development Goals [SDGs]). Ultimately, these goals reflect that much still needs to be done in the least developed settings to ensure entire populations experience equitable life chances, while disability-specific declarations highlight the need for additional efforts so that the most marginalised and excluded in these societies are not left behind [6]. However, where all members of a community face hardships, little is known the experiences of persons with disabilities vis a vis their non-disabled counterparts. Understanding these experiences will help better target interventions and reduce extant inequalities and exclusion.

In relation, there has been little comparison of how people with and without disabilities perceive the quality of their lives across key dimensions, and how closely this corresponds to their material living standards. These facets, involving both objective (i.e. material standards) and subjective (i.e. satisfaction with the quality of these material standards) components, can be understood according to a multidimensional wellbeing framework, where wellbeing can be thought of as broader than that produced by the outcomes of well-functioning economic systems, encompassing the breadth of life experiences of individuals and their households [7]. Consequently, this study aimed to shed light on the extent of perceived relative inequality associated with disability in Liberia by comparing the multidimensional wellbeing of people with and without disabilities across and within households in Liberian communities. We were interested in understanding not only potential differences in material standards that disabled and non-disabled Liberians experience, but also whether perceptions (e.g., satisfaction) with these standards were different. Thus, measuring multidimensional wellbeing, with its focus on objective and subjective components, allows us to detect situations where, even with no gap present in material terms(e.g., in level of income) people with disabilities perceive themselves as worse off (i.e. are less satisfied with their lives) relative to people without disabilities. Moreover, we set out to conduct comparisons between disabled and non-disabled members in the same and other similar households, in order to identify perceived relative inequalities that operate both between and within households as well as those inequalities present in only one context.

### Multi-dimensional wellbeing

In the context of sustainable development, multi-dimensional wellbeing has received increasing attention from both policymakers and development institutions in recent years (e.g., the Human Development Reports; http://hdr.undp.org). A key reason for this is that the areas elucidated by multidimensional wellbeing frameworks are universally valued, meaning that their empirical assessment allows for a degree of understanding in terms of how individuals are prospering on a global scale, who is being left behind, and in what domains [8, 9]. Furthermore, assessment of multidimensional wellbeing using domain-specific indicators allows for a targeted understanding of exactly by which dimensions of life social groups differ, consequently revealing where inequality is located within societies and crucially, how to respond to it [10]. Indeed, although the SDGs are policy commitments and not legally binding, they have reinforced interest in multidimensional wellbeing as they cover many dimensions of life that are traditionally included in wellbeing indexes, such as education, health, employment and political participation [11]. Moreover, they also reiterate links to equitable wellbeing through the ‘leave no one behind’ agenda.

Contemporary conceptualisations of wellbeing recognise that wellbeing is influenced not only by a person’s material living standards, but also by the psychological states that a person forms in relation to these living standards [12, 13]. That is, objective standards such as good quality healthcare, access to an education or a crime-free life are important to multidimensional wellbeing, but so are people’s psychological states, and their subjective perceptions, which may be grounded in personal, social, cultural and religious values. For example, some studies have suggested that subjective life satisfaction is associated with reduced mortality [8, 14], and that domain-specific satisfaction is also associated with more positive outcomes in that area (e.g., job satisfaction leading to better job performance [15]). Evidence also indicates that subjective wellbeing is qualitatively distinct from objective wellbeing. For instance, although studies show that income is positively correlated with subjective wellbeing [16, 17], this association has been shown to be non-linear. For example, Howell and Howell [18] find a stronger relationship between income and subjective wellbeing in low- and middle-income countries while a study by Jebb, Tay, Diener and Oishi [19] suggests that globally there is a satiation point for association between income and subjective wellbeing |(i.e. life evaluation, emotional wellbeing), after which a rise in income is no longer associated with a shift in wellbeing. Taken together, evidence from this body of work shows that multiple objective and subjective elements and multiple dimensions are important to an individual’s overall wellbeing and life chances, necessitating a broad focus across these areas to more comprehensively assess the wellbeing of individuals, including people with and without disabilities.

### Disability and global health

Disability is a complex, dynamic, multifaceted and contested concept, which has been seen through differing perspectives [4]. The internationally accepted biopsychosocial model adopted by the World Health Organisation [20] acknowledges that ‘disability’ is created through the interaction between a person’s impairment and contextual factors, such as their environment and other aspects of their person [4]. Given that disability is partly shaped by such social factors, the marginalisation that people with disabilities experience is a human rights issue necessitating action, rather than a purely medical concern [21, 22]. The influence of external factors on disability and the consequent marginalisation that being disabled brings also means that a disabled person’s wellbeing, including their overall health, is to an extent socially determined [23]. For example, people with disabilities have higher unmet health needs than people without disabilities, especially within low- and middle-income countries [4]. This is partly because people with disabilities are disproportionally more likely to live in poverty and poverty is both a cause and consequence of ill health and, by extension, disability [6] while also posing a pervasive barrier toward accessing medical services (e.g., due to restricted access to transport to healthcare centres [24, 25]). Extant evidence also suggests that people with disabilities have poorer access to education [26] and employment [27] and are at risk of social exclusion within their communities [28, 29], compared to people without disabilities, and that such disadvantages are generally more pronounced in the global South [30].

As countries undergo socioeconomic development, people with disabilities tend to report greater multidimensional wellbeing (i.e. as measured through access to healthcare, education and so forth) where they are able to benefit from inclusive policies and interventions that help bridge existing gaps with non-disabled people [31]. However, in the very poorest settings, it cannot be routinely assumed that the wellbeing of people with disabilities will be worse than people without disabilities across every dimension, because the majority of people from both groups are living in extreme poverty, without good access to services or resources [31]. Even in these settings though, the wellbeing of people with disabilities is adversely affected by other barriers, which are largely not resource dependent, such as disability stigma and discrimination [4, 30]. In sum, it is plausible that the gap in multidimensional wellbeing between people with and without disabilities in the very poorest countries like Liberia can vary within each constituent wellbeing dimension and across its objective and subjective facets. Moreover, just as the global literature has identified that people with disabilities are more likely to experience marginalisation in specific dimensions of wellbeing (e.g., disabled women and income; [32]); in countries where extreme poverty is widespread and pervasive, some individuals with disabilities may be more at risk of inequality relative to people without disabilities. Despite these possibilities, there has been very little empirical investigation into the multidimensional wellbeing of people with and without disabilities experienced within extremely poor settings. The inherent assumption of this is that all poor people experience poverty in the same way, ergo, responses to alleviate poverty can be the same for everyone. This is clearly not the case, and so the present paper aims to shed light on these nuances–and the gaps–in the context of Liberia.

### Multidimensional wellbeing and disability in Liberian communities

Liberia is one of the poorest countries in the world and is currently ranked near the very bottom of the Human Development Index. In addition to nearly a decade of civil war in the 1990’s, the recent Ebola outbreak (2014–2015) also greatly set back recent development efforts to improve living standards. Data collected by the United Nations [33] indicate that 70% of the country’s 4.5 million inhabitants live in poverty and experience low levels of income, health and education, amongst other inequalities. Furthermore, although direct research on wellbeing within Liberian communities is generally quite sparse, what evidence is available suggests it is poor [34, 35].

Amidst the numerous challenges that people in Liberia face, people with disabilities have received relatively little attention compared to other groups identified as in need of support (e.g., youth [36]) and there are little data available on the lives of disabled Liberians, in part due to the absence of good quality national mechanisms to capture it. This is concerning, as without the ability to identify the areas in which Liberians with disabilities perceive and experience inequality compared to those without disabilities, it is difficult to develop targeted responses to redress it.

### Research objectives

To our knowledge, no study has substantively investigated the extent that perceived relative inequality is associated with disability in Liberia, despite a pressing need to do so. In light of this, the present paper compares the multidimensional wellbeing of people with and without disabilities across and within households in Liberian communities through a dedicated household survey. Specifically, our study has two aims:

1. Compare multi-dimensional well-being of disabled people with non-disabled people in Liberia
2. Assess multi-dimensional well-being within households in Liberia

The present paper forms part of a broader mixed methods study in which qualitative interview data were also collected from disabled Liberians regarding their experience of inequality and wellbeing.

## Method

### Ethical statement

This research was approved by University College London’s Research Ethics Committee (1661/006) and ethical approval was also gained from the University of Liberia’s ethics review board. Written consent forms were obtained for all participants.

### Study setting

This study was conducted in Liberia in 2016. Pilot and cognitive testing of the survey took place in the capital city Monrovia, while data collection was carried out in five surrounding counties (Cape Mount, Lofa, Grand Bassa, Monserrado, and Sinoe), purposively chosen to best represent the five geographical regions of the country.

### Questionnaire development

In order to capture both objective and subjective dimensions of wellbeing across multiple domains, we drew on questions used as part of the OECD wellbeing indicators [37] and the Personal Wellbeing Index [38]. To understand the most important dimensions of wellbeing to Liberians and inform our questionnaire development, we also undertook two focus group discussions and two workshops with adult males and females with disabilities in two selected locations (EC and MK). In addition to the verbal accounts collected as part of the workshops, pictorial representations were also presented in each domain of wellbeing (e.g., a picture of a school for education) and their meaning and implication discussed by focus group participants.

The interview-administered questionnaire was tested in a training workshop located in Monrovia with 12 prospective interviewers and piloted on ~12 disabled and ~12 non-disabled people. Based on feedback, we revised accordingly to ensure comprehensible wording, especially for the Likert rating scales, and coherence and consistency within and between domains. The final questionnaire contained the following domains: household details and asset index; health and health services, including the Washington Group questions on disability; Ebola (added following the outbreak in 2014); education, literacy and numeracy; work and employment; transport; social and community interactions; crime and safety; and political engagement.

### Procedure

To understand multidimensional wellbeing among people with and without disabilities in Liberian communities, we sampled households (i.e. families) with a disabled person and neighbouring ‘control’ households (i.e. without a disabled person) across all five study counties. Households with a disabled person were identified by random selection from lists provided by the national umbrella Disabled Person’s Organisation (DPO; National Union of Organisations of the Disabled–NUOD). Control households were selected by choosing the next nearest household to households with disabled members that had eligible individuals available to be interviewed. Given that household heads in many African communities, including Liberia, tend to exert control over household decisions [39] which may have an impact on certain dimensions of wellbeing, we sought to disaggregate our data by also explicitly comparing the wellbeing of household heads and other family members within each type of household. Specifically, in the disabled households we surveyed the household head, a disabled person, and one other (non-disabled) person selected via a random number list after listing all members of the household. In the control households we surveyed the household head, and one other non-disabled person, selecting the latter through matching as closely as possible by age and sex to the disabled person in the disabled household.

The survey was conducted in 2016 by three teams of interviewers using paper questionnaires, following a 5-day training in Monrovia by TC, EC and RN. One of the interviewers on each team was a supervisor who also checked the completion of their team’s questionnaires, and all three teams were supervised by RN, with overall oversight by MK.

### Analytic strategy

Our sample is divided into six groups of people, across households with and without disabled people. The first four of these six groups were individuals living within disabled households:

1. Household head and non-disabled
2. Disabled person
3. Other (non-disabled) person
4. Household head and disabled

The additional two groups were composed on individuals living within non-disabled households divided into:

1. Household head
2. Other person, matched with disabled persons on age and sex

As the aim of our study was to compare measures of wellbeing among people with and without disabilities in Liberia across multiple dimensions, we elected to analyse all Likert-type questions in our household survey, as well as a selection of other questions which corresponded to key indicators in each domain of interest (e.g., health, education; see S1 File for full survey and an overview of questionnaire development). We group these into three clusters in the presentation of our main analyses, acknowledging that all are important to wellbeing: “subjective” indicators (i.e. all questions asking about satisfaction in our survey); “objective” indicators (i.e. questions representing resources or capabilities in the domains, related to an individuals’ basic needs); and “community relations” indicators (i.e. questions pertaining to community participation and social relations, which included indicators that could be seen as both objective and/or subjective). These analyses are presented below in tables, including the wording of each question and scale anchors. All Likert-type questions not falling into these clusters are presented in Table F in S2 File.

To address our two study aims, we undertook four main comparisons:

1. Disabled vs. age and sex matched person from other household (group 2 vs. 6, or if disabled is household head: group 4 vs. 6).
2. Within disabled household: disabled compared to other (group 2 vs. 3, or 4 vs. 3).
3. Within disabled household: disabled compared to household head (group 2 vs. 1).
4. Within non-disabled household: household head compared to other (group 5 vs. 6).

Each of our comparisons was undertaken using a multi-level regression model adjusted for age, sex and education at individual level, and wealth quintile at household level, and clustered by household, village and county. Household wealth quintile was calculated via a principle components analysis of 21 household assets (S1 File). We used linear regression for 5-point Likert scale questions, with coefficients representing the change in score across the 5-point scale, and logistic regression for binary outcomes. Given that we perform multiple comparisons, we apply the Bonferroni correction to our analyses [40]. In comparisons A and B, we conducted 54 respective comparisons, giving a rounded adjusted α of .001, while for comparisons C and D there were 27 comparisons giving a rounded adjusted α of .002.

## Results

### Sample characteristics

A total of 485 disabled and 538 non-disabled households were surveyed, with two thirds of the household heads in disabled households being DPO-list disabled persons themselves (*N* = 330 out of 485 households; Table 1). There were more (74%) female respondents in group 3 (other non-disabled in disabled household) and less (34%) in group 4 (household head and disabled) but otherwise each group comprised equal proportions of males and females. Educational achievement was low across all groups, with 42–52% possessing no formal education, though only 6% of disabled respondents who were not household heads had completed secondary education, compared to 10–15% of the other groups (Table 1). More of the non-disabled households were in the lowest (poorest) wealth quintile, than the disabled households.

**Table 1:** Sample characteristics.

Household	Disabled household (n = 485)				Non-disabled household (n = 538)	
Respondent Type	1. Head of household	2. Disabled	3. Other person	4. Head of household and Disabled	5. Head of household	6. Matched with disabled on age and sex
	n (%)	n (%)	n (%)	n (%)	n (%)	n (%)
Total	155 (100%)	165 (100%)	348 (100%)	330 (100%)	538 (100%)	484 (100%)
Sex^a^: male	74 (47.7%)	69 (41.8%)	92 (26.4%)	219 (66.4%)	274 (50.9%)	218 (45.0%)
female	81 (52.3%)	96 (58.2%)	256 (73.6%)	111 (33.6%)	264 (49.1%)	266 (55.0%)
Age: mean (SD,;min, max)	47.2	37.5	33.6	49.6	49.2	43.4
	(13.5; 21, 97)	(20.3; 17, 93)	(13.9; 17, 92)	(16.0; 18, 93)	(13.1; 20, 90)	(16.9; 17, 90)
Education^b^:						
No formal education	75 (49.0%)	82 (49.7%)	144 (42.1%)	144 (44.2%)	278 (52.3%)	227 (46.9%)
Some primary	21 (13.7%)	38 (23.0%)	64 (18.7%)	55 (16.9%)	57 (10.7%)	66 (13.6%)
Completed primary	6 (3.9%)	4 (2.4%)	5 (1.5%)	7 (2.2%)	14 (2.6%)	29 (6.0%)
Some secondary	23 (15.0%)	27 (16.4%)	83 (24.3%)	59 (18.1%)	73 (13.7%)	85 (17.6%)
Completed secondary	20 (13.1%)	10 (6.1%)	33 (9.7%)	41 (12.6%)	77 (14.5%)	57 (11.8%)
Some college	4 (2.6%)	3 (1.8%)	7 (2.1%)	6 (1.8%)	13 (2.4%)	10 (2.1%)
Completed college	2 (1.3%)	1 (0.6%)	0 (0.0%)	6 (1.8%)	10 (1.9%)	4 (0.8%)
Some university	1 (0.7%)	0 (0.0%)	3 (0.9%)	3 (0.9%)	4 (0.8%)	4 (0.8%)
University	1 (0.7%)	0 (0.0%)	3 (0.9%)	5 (1.5%)	6 (1.1%)	2 (0.4%)
Wealth quintile^c^:						
q1 (poorest)		124 (13.5%)			301 (30.0%)	
q2		213 (23.2%)			205 (20.4%)	
q3		217 (23.6%)			127 (12.7%)	
q4		203 (22.1%)			152 (15.2%)	
q5 (richest)		162 (17.6%)			218 (21.7%)	
County^d^:						
Grand Bassa	27 (17.4%)	25 (15.2%)	39 (11.2%)	64 (19.4%)	81 (15.1%)	78 (16.1%)
Grand Cape Mount	24 (15.5%)	25 (15.2%)	57 (16.4%)	80 (24.2%)	104 (19.3%)	106 (21.9%)
Lofa	47 (30.3%)	51 (30.9%)	86 (24.7%)	71 (21.5%)	120 (22.3%)	120 (24.8%)
Montserrado	8 (5.2%)	12 (7.3%)	88 (25.3%)	56 (17.0%)	122 (22.7%)	69 (14.3%)
Sinoe	49 (31.6%)	52 (31.5%)	78 (22.4%)	59 (17.9%)	111 (20.6%)	111 (22.9%)

All percentages are of non-missing data

^a^ 1 missing data on Sex: Respondent Type (n missing): 1. (0), 2. (0), 3. (0), 4. (1), 5. (0), 6 (0)

^b^ 19 missing data on Education: Respondent Type (n missing): 1. (2), 2. (0), 3. (6), 4. (5), 5. (6), 6 (0)

^c^ 98 missing data on Wealth quintile. Wealth index was only collected at the household level.

^d^ 1 missing data on County: Respondent Type (n missing): 1. (0), 2. (0), 3. (0), 4. (1), 5. (0), 6 (0)

### Inter-household comparisons

Addressing our first aim, we compared both disabled respondents (group 2) and disabled household heads (group 4) to non-disabled respondents (i.e. of non-disabled households; group 6).

For the subjective indictors (see Table 2, columns 2 & 3), we found that both disabled respondents (group 2) and disabled household heads (group 4) reported significantly lower satisfaction with their life, living standards, health, and all aspects of safety (personal, household, & community), compared to the non-disabled respondents (group 6).

**Table 2:** Associations between subjective satisfaction questions and respondent type, adjusted for age, sex, education and wealth quintile, and clustering by household, village and county (regression coefficient, 95%CI, p-value).

Comparison	A (ref group: 6. age and sex matched in non-disabled household)		B (ref group: 3. Other non-disabled in disabled household)		C (ref group: 1. head of household in disabled household)	D (ref group: 6. age and sex matched in non-disabled household)
Question ^a^	2. Disabled	4. Head of household and Disabled	2. Disabled	4. Head of household and Disabled	2. Disabled	5. Head of household (non-disabled house)
*A5*: *Life satisfaction*: Thinking about your own life and personal circumstances, how satisfied are you with your life as a whole?	-0.9858	-0.712	-0.854	-0.58	-1.033	0.29
	(-1.212, -0.760)	(-0.885, -0.538)	(-1.091, -0.617)	(-0.776, -0.383)	(-1.287, -0.780)	(0.148, 0.432)
	*p*<0.0001	*p*<0.0001	*p*<0.0001	*p*<0.0001	*p*<0.0001	*p*<0.0001
*B2_1*: *Living standards*: How satisfied are you with your own standard of living?	-0.943	-0.62	-0.897	-0.574	-0.687	0.334
	(-1.169, -0.718)	(-0.795, -0.444)	(-1.132, -0.662)	(-0.770, -0.377)	(-0.942, -0.433)	(0.197, 0.483)
	*p*<0.0001	*p*<0.0001	*p*<0.0001	*p*<0.0001	*p*<0.0001	*p*<0.0001
*C1*: *Health*: How satisfied are you with your health overall?	-1.334	-1.157	-1.568	-1.39	-1.384	-0.118
	(-1.578, -1.090)	(-1.347, -0.966)	(-1.824, -1.311)	(-1.609, -1.171)	(-1.675, -1.094)	(-0.280, 0.044)
	*p*<0.0001	*p*<0.0001	*p*<0.0001	*p*<0.0001	*p*<0.0001	*p* = 0.153
*C4_1*: *Health access*: How satisfied are you with your access to health services?	-0.19	-0.463	-0.707	-0.98	-0.452	0.175
	(-0.422, 0.042)	(-0.643, -0.283)	(-0.948, -0.467)	(-1.181, -0.780)	(-712, -0.192)	(0.029, 0.321)
	*p* = 0.109	*p*<0.0001	*p*<0.0001	*p*<0.0001	*p* = 0.001	*p* = 0.019
*C4_13*: *Health* care: How satisfied are you with the health care you receive?^b^	-0.106	-0.198	-0.531	-0.623	-0.325	0.173
	(-0.326, 0.110)	(-0.366, -0.030)	(-0.755, -0.307)	(-0.809, -0.437)	(-0.565, -0.855)	(0.037, 0.309)
	*p* = 0.337	*p* = 0.021	*p* < 0.001	*p*<0.0001	*p* = 0.008	*p* = 0.013
*D3_1*: *Education*: How satisfied are you with the education/ school in your community?	0.826	0.446	0.058	-0.438	0.551	0.382
	(0.576, 1.075)	(0.254, 0.638)	(-0.316, 0.199)	(-0.655, -0.221)	(-0.231, 0.341)	(0.218, 0.545)
	*p*<0.0001	*p*<0.0001	*p* = 0.658	*p*<0.0001	*p* = 0.706	*p*<0.0001
*E1_11*: *Work*: How satisfied are you with your work/employment?	0.0512	-0.395	-0.216	-0.662	0.078	0.211
	(-0.418, 0.521)	(-0.700, -0.089)	(-0.689, 0.257)	(-1.003, -0.321)	(-0.434, 0.589)	(-0.016, 0.437)
	*p* = 0.831	*p* = 0.011	*p* = 0.370	*p*<0.0001	*p* = 0.767	*p* = 0.068
*F1_1*: *Transport*: How satisfied are you with the access to transport in your community?	-0.492	-0.045	-0.484	-0.036	-0.908	0.495
	(-0.733, -0.251)	(-0.233, 0.143)	(-0.738, -0.229)	(-0.254, 0.181)	(-1.193, -0.624)	(0.335, 0.656)
	*p*<0.0001	*p* = 0.640	*p*<0.0001	*p* = 0.744	*p*<0.0001	*p*<0.0001
*G2_1*: *Relationships with Friends*: How satisfied are you with your relationships with friends?	-0.256	-0.174	-0.099	0.017	-0.826	-0.176
	(-0.384, -0.129)	(-0.273, -0.076)	(-0.235, 0.037)	(-0.133, 0.098)	(-0.231, 0.073)	(-0.262, -0.090)
	*p*<0.0001	*p* = 0.001	*p* = 0.154	*p* = 0.771	*p* = 0.298	*p*<0.0001
*G2_2*: *Relationships with Household*: How satisfied are you with your relationships with your household?	-0.109	-0.237	-0.037	-0.165	0.041	-0.175
	(-0.217, -0.000)	(-0.322, -0.152)	(-0.152, 0.078)	(-0.265, -0.066)	(-0.089, 0.170)	(-0.248, -0.102)
	*p* = 0.049	*p*<0.0001	*p* = 0.529	*p* = 0.001	*p* = 0.541	*p*<0.0001
*G2_3*: *Relationship with Partner*: How satisfied are you with your relationship with your husband/wife/partner?	-0.133	-0.263	0.172	0.042	0.0003	-0.178
	(-0.363, 0.098)	(-0.408, -0.118)	(-0.066, 0.410)	(-0.126, 0.209)	(-0.247, 0.247)	(-0.292, -0.065)
	*p* = 0.259	*p*<0.0001	*p* = 0.157	*p* = 0.624	*p* = 0.998	*p* = 0.002
*H4_1*: *Personal Safety*: How satisfied are you with your personal safety?	-0.579	-0.667	-0.336	-0.424	-0.229	-0.88
	(-0.775, -0.383)	(-0.819, -0.515)	(-0.542, -0.129)	(-0.600, -0.248)	(-0.462, 0.003)	(-1.010, -0.750)
	*p*<0.0001	*p*<0.0001	*p* = 0.001	*p*<0.0001	*p* = 0.053	*p*<0.0001
*H4_2*: *Household Safety*: How satisfied are you with the safety of your household?	-0.287	-0.365	-0.142	-0.22	-0.077	-0.26
	(-0.429, -0.145)	(-0.475, -0.255)	(-0.291, 0.007)	(-0.345, -0.094)	(-0.241, 0.088)	(-0.352, -0.167)
	*p*<0.0001	*p*<0.0001	*p* = 0.061	*p* = 0.001	*p* = 0.360	*p*<0.0001
*H4_3*: *Community Safety*: How satisfied are you with the safety of your community?	-0.31	-0.47	-0.221	-0.381	-0.013	-0.375
	(-0.472, -0.149)	(-0.595, -0.345)	(-0.390, -0.052)	(-0.523, -0.239)	(-0.199, 0.173)	(-0.479, -0.271)
	*p*<0.0001	*p*<0.0001	*p* = 0.010	*p*<0.0001	*p* = 0.893	*p*<0.0001

^a^ All questions are asked as a 1–5 Likert scale: 1 = Not at all satisfied; 2 = a bit unsatisfied; 3 = not satisfied or unsatisfied; 4 = a bit satisfied; 5 = completely satisfied

Given multiple comparisons *p*-values above 0.001 are not considered significant for comparisons A and B and above 0.002 for comparisons C and D.

^b^ Regression models did not converge, though for all models log likelihood remained unchanged (backed up) from 8^th^ iteration to 100^th^ iteration.

Compared to the matched non-disabled respondents, disabled respondents also reported significantly less satisfaction with transport and relationships with friends, while disabled household heads were significantly less satisfied with health access and relationships with both their household and partner. However, both disabled respondents and disabled household heads reported significantly greater satisfaction with education, compared to the matched non-disabled respondent.

All other comparisons were non-significant (i.e. *p* > .001). As such, there was no difference in satisfaction with healthcare received between the matched non-disabled respondents and either the disabled respondents or disabled household heads.

For the objective indictors (Table 3, columns 2 & 3), we found that disabled respondents (group 2), but not disabled household heads (group 4), reported less access to transport and lower voter participation compared to the matched non-disabled respondents. Conversely, compared to the non-disabled respondents, disabled household heads reported getting healthcare more frequently when needed and significantly more experience of crime.

**Table 3:** Associations between objective questions and respondent type, adjusted for age, sex, education and wealth quintile, and clustering by household, village and county (regression coefficient, 95%CI, p-value).

Question ^a^	A (ref group: 6. age and sex matched in non-disabled household)		B (ref group: 3. Other non-disabled in disabled household)		C (ref group: 1. head of household in disabled household)	D (ref group: 6. age and sex matched in non-disabled household)
	2. Disabled	4. Head of household and Disabled	2. Disabled	4. Head of household and Disabled	2. Disabled	5. Head of household (non-disabled house)
*C4_2*: *Getting needed Healthcare*: How often can you get the healthcare you need?^a^	-0.224	0.373	-0.109	0.488	-0.839	0.138
	(-0.364, -0.085)	(0.264, 0.482)	(-0.257, 0.038)	(0.363, 0.614)	(-1.004, -0.674)	(0.046, 0.231)
	*p* = 0.002	*p*<0.0001	*p* = 0.146	*p*<0.0001	*p*<0.0001	*p* = 0.003
*D1*: *Education*: What is the highest level of education you have completed? ^b^	-0.335	0.018	-0.338	0.015	-0.516	0.078
	(-0.629, -0.041)	(-0.212, 0.247)	(-0.644, -0.032)	(-0.239, 0.268)	(-0.842, -0.190)	(-0.106, 0.262)
	*p* = 0.025	*p* = 0.880	*p* = 0.030	*p* = 0.909	*p* = 0.002	*p* = 0.407
*E1_4*: *Income*: How much money do you make per month? (Liberian $)	-1087	-1812	641	-84	-8106	5538
	(-9232, 7058)	(-7522, 3898)	(-7334, 8615)	(-6279, 6110)	(-17457, 1246)	(1444, 9632)
	*p* = 0.794	*p* = 0.534	*p* = 0.875	*p* = 0.979	*p* = 0.089	*p* = 0.008
*F1_3 Transport Access*: How often do you have access to the transport you need? ^a^	-0.702	-0.039	-0.476	0.186	-1.051	0.031
	(-0.873, -0.532)	(-0.172, 0.093)	(-0.657, -0.296)	(0.032, 0.341)	(-1.254, -0.848)	(-0.083, 0.145)
	*p*<0.0001	*p* = 0.560	*p*<0.0001	*p* = 0.018	*p*<0.0001	*p* = 0.595
*G4_1 Vote*: Do you vote? ^c^	-0.251	0.002	-0.159	0.093	-0.288	0.01
	(-0.300, -0.201)	(-0.037, 0.041)	(-0.213, -0.106)	(0.048, 0.138)	(-0.347, -0.229)	(-0.024, 0.043)
	*p*<0.0001	*p* = 0.915	*p*<0.0001	*p*<0.0001	*p*<0.0001	*p* = 0.574
*H1_3 & H1_5*: *Crime*: Have you personally experienced any form of crime or violence in the last year? Has anyone in your household witnessed any crime or violence in the last year? ^d^	0.131	0.19	0.02	0.079	-0.036	0.11
	(0.044, 0.218)	(0.124, 0.256)	(-0.072, 0.113)	(0.003, 0.155)	(-0.136, 0.063)	(0.050, 0.170)
	*p* = 0.003	*p*<0.0001	*p* = 0.664	*p* = 0.042	*p* = 0.477	*p*<0.001

^a^ Coded on a 4 point scale: 1 = Never; 2 = Occasionally/Sometimes; 3 = Most of the time; 4 = All of the time

^b^ Coded on a 9-point scale: 1 = No formal education; 2 = Some primary; 3 = Completed primary; 4 = Some secondary; 5 = Completed secondary; 6 = Some college; 7 = Completed college; 8 = Some university; 9 = University. Note this model, with education as the outcome, unlike the other models obviously did not include education as an explanatory variable.

^c^ 1 = Yes (sometimes or always); 0 = No; the two respondents who refused the question were coded as missing

^d^ 1 = Yes (once, or more than once for personally experienced crime, or household member witnessed a crime); 0 = No (not experienced crime in the past year); ‘don’t know’ (88; 7%) and ‘refused answer’ (99; 3%) to question H1_3 recoded as missing

Given multiple comparisons *p*-values above 0.001 are not considered significant for comparisons A and B and above 0.002 for comparisons C and D.

All other comparisons were non-significant (i.e. p > .001). As such, there was no difference in education level or income between the matched non-disabled respondents and either the disabled respondents or disabled household heads.

For the community relations indictors (Table 4, columns 2 & 3), our findings show that both disabled respondents (group 2) and disabled household heads (group 4) felt less included in the community, were less likely to engage in community participation, held less trust in neighbours and felt less included in decision making compared to the matched non-disabled respondents.

**Table 4:** Associations between community relations indicators and respondent type, adjusted for age, sex, education and wealth quintile, and clustering by household, village and county (regression coefficient, 95%CI, p-value).

Question	A (ref group: 6. age and sex matched in non-disabled household)		B (ref group: 3. Other non-disabled in disabled household)		C (ref group: 1. head of household in disabled household)	D (ref group: 6. age and sex matched in non-disabled household)
	2. Disabled	4. Head of household and Disabled	2. Disabled	4. Head of household and Disabled	2. Disabled	5. Head of household (non-disabled house)
*G1_1*: *Community Inclusion*: How included do you feel in your community?^a^	-0.8	-0.672	-0.633	-0.505	-0.519	-0.224
	(-1.041, -0.560)	(-0.859, -0.484)	(-0.887, -0.379)	(-0.721, -0.289)	(-0.803, -0.235)	(-0.383, -0.065)
	*p*<0.0001	*p*<0.0001	*p*<0.0001	*p*<0.0001	*p*<0.001	*p* = 0.006
*G1_3*: *Community Participation*: Do you participate in any community activities? ^b^	-0.414	-0.164	-0.342	-0.093	-0.368	-0.037
	(-0.496, -0.332)	(-0.229, -0.100)	(-0.430, -0.255)	(-0.168, -0.017)	(-0.467, -0.269)	-0.092, 0.185
	*p*<0.0001	*p*<0.0001	*p*<0.0001	*p* = 0.016	*p*<0.0001	*p* = 0.192
*G1_6*: *Friends*: Do you have friends?^c^	-0.121	0.068	-0.241	-0.052	-0.303	-0.026
	(-0.210, -0.032)	(-0.001, 0.137)	(-0.334, -0.148)	(-0.132, 0.027)	(-0.408, -0.199)	(-0.084, 0.033)
	*p* = 0.008	*p* = 0.053	*p*<0.0001	*p* = 0.199	*p*<0.0001	*p* = 0.387
*G3_1 & G3_3*: *Getting Help from Community*: Do your neighbours help you when you ask for assistance? Does your community help when you ask for assistance?^d^	-0.142	-0.021	-0.392	-0.271	-0.269	0.058
	(-0.278, -0.006)	(-0.127, 0.084)	(-0.534, -0.249)	(-0.391, -0.151)	(-0.425, -0.112)	(-0.030, 0.146)
	*p* = 0.041	*p* = 0.693	*p*<0.0001	*p*<0.0001	*p* = 0.001	*p* = 0.194
G3_2 & G3_4: *Giving Help to Community*: Do you help your neighbours when they ask for assistance? Do you help your community in community initiatives?^d^	-0.354	-0.039	-0.57	-0.256	-0.601	0.084
	(-0.486, -0.223)	(-0.141, 0.062)	(-0.709, -0.431)	(-0.373, -0.138)	(-0.756, -0.446)	(-0.002, 0.171)
	*p*<0.0001	*p* = 0.447	*p*<0.0001	*p*<0.0001	*p*<0.0001	*p* = 0.055
G3_5: *Trust*: How much do you trust your neighbours?^e^	-0.448	-0.302	-0.225	-0.078	-0.21	-0.122
	(-0.635, -0.261)	(-0.446, -0.158)	(-0.423, -0.026)	(-0.245, 0.088)	(-0.429, 0.010)	(-0.244, -0.001)
	*p*<0.0001	*p*<0.0001	*p* = 0.026	*p* = 0.355	*p* = 0.061	*p* = 0.048
*G4_3*: *Inclusion in Decision Making*: How included do you feel in the decision making of your community?^a^	-1.131	-0.908	-0.934	-0.71	-0.937	-0.235
	(-1.384, -0.879)	(-1.103, -0.713)	(-1.200, -0.668)	(-0.933, -0.487)	(-1.230, -0.644)	(-0.398, -0.071)
	*p*<0.0001	*p*<0.0001	*p*<0.0001	*p*<0.0001	*p*<0.0001	*p* = 0.005

^**a**^ Coded on a 5 point scale: 1 = Not included at all; 2 = A bit not included; 3 = Neither included nor not included; 4 = A bit included; 5 = Very included

^b^ 1 = Yes; 0 = No

^c^ 1 = Many (Yes, many/enough); 0 = Not many (Yes, a few/not enough or No)

^d^ Average score of the two items; coded on a 4 point scale: 1 = Never; 2 = Not often; 3 = Most of the time; 4 = All the time

^e^ Coded on a 5 point scale: 1 = Not at all; 2 = Not very much; 3 = No opinion; 4 = A bit; 5 = Completely

Given multiple comparisons *p*-values above 0.001 are not considered significant for comparisons A and B and above 0.002 for comparisons C and D.

Disabled respondents, but not disabled household heads, also reported giving less help to the community compared to the matched non-disabled respondents. All other comparisons were non-significant (i.e. *p* > .001), meaning that there was no difference between the matched non-disabled respondents and either the disabled respondents or disabled household heads in amount of friends or level of help received from the community.

### Intra-household comparisons

We computed three sets of intra-household comparisons to comprehensively address our second aim. In the first set, we compared both disabled respondents (group 2) and disabled household heads (group 4) to non-disabled respondents within the same household (group 3).

For the subjective indictors in this first set of comparisons (see Table 2, columns 4 & 5), we found that both disabled respondents (group 2) and disabled household heads (group 4) reported lower satisfaction with their life, living standards, overall health, healthcare access, and healthcare, compared to non-disabled members of their household (group 3).

Compared to these non-disabled respondents, disabled respondents also reported significantly less satisfaction with transport, while disabled household heads were significantly less satisfied with education, work and personal and community safety.

All other comparisons were non-significant (i.e. *p* > .001). This means there were no differences in satisfaction regarding friend, household or partner relationships, as well as household safety between non-disabled members of the household and either disabled respondents or disabled household heads.

For the objective indictors, this first set of intra-household comparisons (Table 3, columns 4 & 5) indicate that disabled respondents (group 2) reported less access to transport and lower voter participation, compared to non-disabled members of the household (group 3), whereas disabled household heads (group 4) reported greater access to healthcare and higher voter participation, compared to these non-disabled respondents.

All other comparisons were non-significant (i.e. *p* > .001). That is, there were no differences in education level, income and experiences of crime between non-disabled members of the household and either disabled respondents or disabled household heads.

For the community relations indictors in this first set of intra-household comparisons (see Table 4, columns 4 & 5), we found that both disabled respondents (group 2) and disabled household heads (group 4), felt less included in the community, received less help from the community, gave less help to the community and felt less included in decision-making.

Disabled respondents, but not disabled household heads, also reported a lower likelihood of engaging in community participation and having fewer friends compared to non-disabled members of their household. All other comparisons were non-significant (i.e. *p* > .001). As such, there was no difference in trust held in neighbours between any of the three groups.

Our second set of intra-household comparisons were between disabled respondents (group 2) and non-disabled household heads in the same households (group 1). For the subjective indicators (see Table 2, column 6), this comparison revealed that disabled respondents reported lower satisfaction with their life, living standards, health, health access, and transport, compared to non-disabled household heads, with differences for all other subjective indicators non-significant (i.e. *p* > .002).

For the objective indicators (see Table 3, column 6), this second set of comparisons revealed that disabled respondents reported less access to healthcare, less access to transport and less voter participation compared to non-disabled household heads. Conversely, there were no differences within income education or experience of crime (i.e. *p* > .002).

For the community relations indicators (see Table 4, column 6), this second set of comparisons showed that, compared to non-disabled household heads, disabled respondents reported lower levels of community participation across all indicators surveyed (i.e. all *p* < .002) except one. Specifically, there was no significant difference in trust in neighbours.

Our third and final set of intra-household comparisons was between household heads in non-disabled households (group 5) and the non-disabled respondents in the same households (group 6). For the subjective indicators (see Table 2, column 7), these non-disabled household heads reported greater satisfaction with their life, living standards, education and transport, but less satisfaction with two aspects of relationships (friendships, household) and all aspects of safety (personal, household, & community) compared to the non-disabled respondents. There were no significant differences in satisfaction with health, health access, healthcare, work and relationship with partner.

For the objective indicators (see Table 3, column 7), the third set of comparisons indicated that non-disabled household heads reported experiencing more crime compared to non-disabled respondents in the same households. There were no significant differences in healthcare access, education, income, transport access, healthcare or voting.

For the community relations indicators (see Table 4, column 7), the third set of comparisons revealed no differences between non-disabled household heads and non-disabled members of the same household on any community participation indicator.

We also cross-checked our findings (i.e. with the DPO-list identified disabled persons) using the Washington Group questions to identify disabled status. Results did not substantively change (see S3 File).

## Discussion

The present research makes an important contribution by shedding light on the areas of perceived relative inequality which are associated with disability within a context where most individuals experience extreme hardship. Specifically, we compared the multi-dimensional wellbeing of people with and without disabilities across and within households in Liberian communities, using statistical models that adjusted for demographic characteristics (age, sex, education & wealth) and clustered by household, village and county. We consider our findings for each of our primary groups of interest in turn (i.e. disabled respondents and disabled household heads).

Our results suggest that disabled Liberians (group 2) experience many inequalities in their multidimensional wellbeing relative to non-disabled Liberians. Compared to both similar non-disabled Liberians in other households (group 6) and non-disabled members of their own household (group 3) disabled Liberians (groups 2) report lower satisfaction with their life, living standards, health, and transport, as well as reduced access to transport and voter participation. Moreover, across all comparisons, these disabled respondents generally had poorer community relations (i.e. felt less included in the community, were less likely to participate, gave less help to the community and felt less included in decision-making). Additionally, the same perceived relative inequalities were observed when disabled respondents (group 2) were compared with the non-disabled heads of their households (group 1). The consistency of these inequalities across the multiple comparisons performed agrees with prior evidence that has highlighted the tendency for people with disabilities to experience marginalisation across many wellbeing dimensions globally [4].

Our findings further qualify which disabled Liberians are at the most risk of marginalisation by illustrating how being a household head helps protect disabled people from experiencing certain inequalities relative to non-disabled people. Specifically, disabled Liberian household heads (group 4) did not perceive reduced access to transport or voter participation compared to non-disabled Liberians in other households (group 6) and reported greater transport access and voter participation that non-disabled members of their own household (group 3). This could be due to the fact that household heads generally possess decision-making power in the household [39]. What these findings highlight overall is the importance of understanding intra-household dynamics, and not assuming that resources (e.g., social protection payments or other welfare support) allocated at the level of the household are equally—or equitably- distributed. They also demonstrate the extent to which non-disabled adults and children within a household with a disabled member can be disadvantaged. In this respect, our findings also indicate that in non-disabled households, individuals have similar levels of transport access and voter participation regardless of whether or not they are a household head (groups 5 and 6). As such, efforts to redress the marginalisation of disabled people in Liberia and similar settings should also consider how the household may have been impacted to meet the goal of no-one left behind.

Despite some apparent protective factors, in some areas disabled household heads may encounter additional marginalisation. For instance, disabled household heads (group 4), but not other disabled Liberians (group 2) reported experiencing more crime relative to non-disabled Liberians in other households (group 6). Moreover, although disabled household heads (group 4) reported less satisfaction with healthcare access and healthcare than non-disabled Liberians in their households (group 3), they also reported being able to get access healthcare when needed more frequently than non-disabled Liberians in their own and also other households (group 3 & 6). This does not correspond to the identified pattern in non-disabled Liberian households, where household heads (group 5) reported no differences in satisfaction related to healthcare or healthcare access (group 6). As such, while disabled household heads may not experience relative inequality in terms of healthcare access, they may nonetheless encounter additional barriers that impact on their satisfaction with the obtained healthcare and equity of outcomes of such healthcare, such as negative attitudes from healthcare providers [41]. This is substantiated by the fact that qualitative data collected from healthcare providers as part of the broader project (to date unpublished) showed that none had received any disability training.

Previous studies have identified that in some respects having a disabled family member can impact the entire household [1]. Interestingly, in one wellbeing dimension assessed in our study, differences were present at the inter-household level, but not the intra-household level. Specifically, disabled Liberians, including household heads (groups 2 & 4) reported less trust in neighbours compared to non-disabled Liberians in other households (group 6), but there were no intra-household differences (i.e. with groups 1 or 3). This is consistent with the notion that these perceived relative inequalities are associated with disability, and may impact on the household, not only the disabled individual. Supporting this interpretation, non-disabled members of disabled households (group 3) reported less trust in neighbours, (B = -.223, *p* = 0.004, LLCI = -0.374, ULCI = -0.073) compared to non-disabled respondents in other households (group 6).

Finally, the comparisons revealed no differences between disabled Liberians (groups 2 & 4) and non-disabled Liberians (groups 1, 3 or 6) in terms of income or education. Moreover, comparisons between disabled and non-disabled household heads (groups 4 and 5) and other members of their households (groups 3 and 6) showed that household heads reported less satisfaction with personal and community safety regardless of their disability status. These findings are consistent with the idea that in the very poorest settings, people may experience broadly similar levels of disadvantage in some areas regardless of their disability status [31]. Given that our findings indicate no gaps in education or income, but that disabled Liberians report poorer life satisfaction and living standards, they are also consistent with prior evidence that economic status is only weakly correlated with subjective wellbeing [18]. Such findings place the onus on disability-inclusive development efforts in Liberia to both *prevent* gaps within the income and educational levels of disabled and non-disabled Liberians from appearing, that is to say, reducing the inequalities from growing in the first place; as well as to close the gaps disabled Liberians experience in key facets of their subjective wellbeing by implementing targeted programmes and policies to do this.

In terms of further practical implications for Liberia, our results suggest the need for interventions that increase life satisfaction and social inclusion among all disabled Liberians and both transport access and political participation among disabled Liberians who are not household heads in particular. There is also a need to understand exactly why satisfaction with healthcare and healthcare access is poor among disabled Liberian household heads who can access it on an equal basis to non-disabled Liberians and report the best level of access in their household. One possible reason might be to do with perceptions of services after the Ebola outbreak during the research period, but overall it warrants further exploration. Further research is also needed to understand why Liberians with disabled household members may report experiencing less trust in their neighbours, compared to Liberians in non-disabled households. This may be related to feeling discriminated against within communities. Finally, across the objective domains in particular, future research should also explore where disabled Liberians experience relative inequity (i.e. in what dimensions disabled and non-disabled Liberians have equal access to a resource, but need more of it to be on the same footing as others in society).

Our research had two limitations of note. Firstly, we identified disabled participants in our study through a list provided by a national Disabled Peoples’ Organisation (DPO). It is plausible that disabled persons in contact with a DPO may possess higher levels of wellbeing in some dimensions (e.g., in terms of their educational attainment, political engagement and community relations) relative to disabled persons who are not. Therefore, although our study identifies many areas of perceived relative inequality between disabled and non-disabled Liberians the magnitude of these gaps for non-DPO listed disabled Liberians may be larger. Secondly, this was a cross-sectional study. As such, while we provide a detailed snapshot of multidimensional wellbeing between disabled and non-disabled Liberians, we are unable to discern how stable the relative inequalities identified are over time, especially given the deleterious impact that Ebola had on Liberian communities and particularly disabled individuals at the time of our study. In this respect, the findings represent a call to action to understand the causes of some of the identified gaps and how to address them as an urgent course of action.

## Conclusion

In sum, our study provides a differentiated understanding of perceived relative inequality between disabled and non-disabled people in a context of extreme hardship and in a country where comparatively little is known about the lives of people with disabilities. We find that while disabled Liberians are managing similarly to non-disabled individuals in key areas of wellbeing, like income and education, there are many aspects of perceived relative inequality that need to be redressed (e.g., life satisfaction, social exclusion). Moreover, disabled Liberians who are not household heads are more at risk of relative inequality in some areas, notably exclusion from accessing transport and political participation. As such, actors concerned with disability-inclusive development in Liberia and other similar settings should pay attention not only to broad development issues, but also to specific dimensions where interventions need to be targeted, acknowledging that these may be beyond material (objective) indicators and reach into more psychological (subjective) aspects of wellbeing. Put differently, given the systemic marginalisation and exclusion experienced by persons with disabilities in Liberia, merely making programmes technically inclusive will not address the broader issues of self-esteem, trust, confidence and wellbeing. This work is also timely, as there are a number of initiatives currently in progress building on advocacy work already underway by disabled people and their representative organisations. These include the development of a National Disability Action Plan [42], which aims to support the delivery of the commitments made by the Government of Liberia. We hope our findings are helpful in guiding these future efforts.

## Supporting information

S1 File Study survey. (DOCX)

S2 File Supplementary statistical results tables. (DOCX)

S3 File Analysis with Washington Group questions. (DOCX)

S4 File Data used in this study. (DTA)
