# Supplementary material for: Inter- and intra-household perceived relative inequality among disabled and non-disabled people in Liberia
Source: PLoS One. 2019 Jul 17;14(7):e0217873. doi: 10.1371/journal.pone.0217873 (PMC6636711; doi:10.1371/journal.pone.0217873)
Supplement: S1 File — (DOCX) [file pone.0217873.s001.docx]

**Respondent Details**

***Before we begin, we would first like to ask you to answer a few general questions about you and your household.***

| **A1** | **Gender *[circle one option]*** | | | | | | | | | | | | | | | | | | | | | | | |  |
| --- | --- | --- | --- | --- | --- | --- | --- | --- | --- | --- | --- | --- | --- | --- | --- | --- | --- | --- | --- | --- | --- | --- | --- | --- | --- |
|  | Male | | | | 1 | | | | | | | Female | | | | | | | | | | | 2 | |  |
| **A2** | **Age at last birthday (years)** | |  | | | | | | | | | | | | | | | | | | | | | |  |
| **A2_1** | **Were you born in [location]? *[circle one option]*** | | | | | | | | | | | | | | | | | | | | | | | |  |
|  | Yes (go to A3) | | 1 | | No (go to A2_2) | | | | | | | | | | | | | | | | | | 2 | |  |
| **A2_2** | **If no, WHERE were you born?** | | | | | | | | |  | | | | | | | | | | | | | | |  |
|  | **If no, WHEN did you move in [location] (year)?** | | | | | | | | | | | | | | | | |  | | | | | | |  |
| **A2_3** | **If no, WHY did you move to [location]? *[circle as many as apply]*** | | | | | | | | | | | | | | | | | | | | | | | |  |
|  | Marriage | | | 1 | | | | | | | Divorce/separation | | | | | | | | 5 | | | | | |  |
|  | Work | | | 2 | | | | | | | Other (please specify) | | | | | | | | 6 | | | | | |  |
|  | Displaced by war | | | 3 | | | | | | | Don’t know | | | | | | | | 88 | | | | | |  |
|  | Ebola | | | 4 | | | | | | | Refused answer | | | | | | | | 99 | | | | | |  |
| *****QUESTIONS A3 to A3_4 ONLY ASK HEAD OF HOUSEHOLD. OTHERWISE GO TO A4_1***** | | | | | | | | | | | | | | | | | | | | | | | | |  |
| **A3** | **Total number in household** | | | | **Adults** | | | | | | |  | | | | | **Children** (under 17) | | | | | |  | |  |
| **A3_1** | **How many rooms are in your household?** | | | | | | | | | | | | | |  | | | | | | | | | |  |
| **A3_2** | **Does the household own the dwelling? *[circle one option]*** | | | | | | | | | | | | | | | | | | | | | | | |  |
|  | Owns the dwelling | | | | | | | | | | | 1 | | | Other (please specify) | | | | | | | | 5 | |  |
|  | Rents the dwelling | | | | | | | | | | | 2 | | | Don’t know | | | | | | | | 88 | |  |
|  | Uses without paying rent | | | | | | | | | | | 3 | | | Refused to answer | | | | | | | | 99 | |  |
|  | Nomadic or temporary | | | | | | | | | | | 4 | | |  | | | | | | | |  | |  |
| **A3_3** | **Does the household own any land? *[circle one option]*** | | | | | | | | | | | | | | | | | | | | | | | |  |
|  | Yes (go to A3_4) | | | | | 1 | | | | | | | | No/don’t know (go to A4_1) | | | | | | | 2 | | | |  |
| **A3_4** | **How many lots does the household own? *[write in number of lots]*** (4 lots = 1 acre) | | | | | | | ________ | | | | | | | Don't know | | | | | -88 | | | | |  |
| **A3_5** | **What is the land used for? *[don’t read the options, circle as many as apply]*** | | | | | | | | | | | | | | | | | | | | | | | |  |
|  | Household lives on the land | | | | | | | | | | | 1 | | | Not used for anything | | | | | | | | 6 | |  |
|  | Farmed by the household | | | | | | | | | | | 2 | | | Used for business purposes | | | | | | | | 7 | |  |
|  | Rented out for farming | | | | | | | | | | | 3 | | | Other (please specify) | | | | | | | | 8 | |  |
|  | Renting out for housing | | | | | | | | | | | 4 | | | Don’t know | | | | | | | | 88 | |  |
|  | Rented for business purposes | | | | | | | | | | | 5 | | | Refused to answer | | | | | | | | 99 | |  |
| **A4_1** | | **Relationship of respondent to the head of household *[circle one option]*** | | | | | | | | | | | | | | | | | | | | | | | |
|  | | Is head of household | | | | | 1 | | | | | | Niece/nephew | | | | | | | | | | | 6 | |
|  | | Spouse | | | | | 2 | | | | | | Grandparent | | | | | | | | | | | 7 | |
|  | | Son/daughter | | | | | 3 | | | | | | Grandchild | | | | | | | | | | | 8 | |
|  | | Brother/sister | | | | | 4 | | | | | | Non-relative/family member | | | | | | | | | | | 9 | |
|  | | Parent | | | | | 5 | | | | | | Other (please specify) | | | | | | | | | | | 10 | |
| **A4_2** | | **Marital** **status *[circle one option]*** | | | | | | | | | | | | | | | | | | | | | | | |
|  | | Single | | | | | 1 | | | | | | Separated | | | | | | | | | | | 6 | |
|  | | Married monogamous | | | | | 2 | | | | | | Divorced | | | | | | | | | | | 7 | |
|  | | Married polygamous | | | | | 3 | | | | | | Widowed / Widower | | | | | | | | | | | 8 | |
|  | | Living together (not married) | | | | | 4 | | | | | | Other (please specify) | | | | | | | | | | | 9 | |
|  | | In a relationship (not living with partner) | | | | | 5 | | | | | |  |  |  |  |  |  |  |  |  |  |  |  | |
| **A4_3** | | **Religion [*circle one option]*** | | | | | | | | | | | | | | | | | | | | | | | |
|  | | Christian | | | | | 1 | | | | | | Other (please specify) | | | | | | | | | | | 4 | |
|  | | Muslim | | | | | 2 | | | | | |  |  |  |  |  |  |  |  |  |  |  |  |  |
|  | | Traditional beliefs | | | | | 3 | | | | | | No religion | | | | | | | | | | | 5 | |
| **A4_4** | | **What is your ethnic affiliation? *[circle one option]*** | | | | | | | | | | | | | | | | | | | | | | | |
|  | | Bassa | | | | | 1 | | | | | | Krahn | | | | | | | | | | | 11 | |
|  | | Belle | | | | | 2 | | | | | | Kru | | | | | | | | | | | 12 | |
|  | | Dey | | | | | 3 | | | | | | Lorma | | | | | | | | | | | 13 | |
|  | | Gbandi | | | | | 4 | | | | | | Mandingo | | | | | | | | | | | 14 | |
|  | | Gbi | | | | | 5 | | | | | | Mano | | | | | | | | | | | 15 | |
|  | | Gio | | | | | 6 | | | | | | Mende | | | | | | | | | | | 16 | |
|  | | Gola | | | | | 7 | | | | | | Sapo | | | | | | | | | | | 17 | |
|  | | Grebo | | | | | 8 | | | | | | Vai | | | | | | | | | | | 18 | |
|  | | Kpelle | | | | | 9 | | | | | | Congo Liberian/Americo-Liberian | | | | | | | | | | | 19 | |
|  | | Kissi | | | | | 10 | | | | | | Naturalised Liberian | | | | | | | | | | | 20 | |
| **A5** | | **Thinking about your own life and personal circumstances, how satisfied are you with your life as a whole? *[read out the options, circle one option]*** | | | | | | | | | | | | | | | | | | | | | | | |
|  | | Not at all satisfied | a bit unsatisfied | | | | | | not satisfied or unsatisfied | | | | | | | a bit satisfied | | | | | | completely satisfied | | | |
|  | | 1 | 2 | | | | | | 3 | | | | | | | 4 | | | | | | 5 | | | |
| **A6** | | **What do you think are the three most important things to live a life with dignity? *[read options, and write 1, 2, 3 next to the chosen ones, where 1 = most important]*** | | | | | | | | | | | | | | | | | | | | | | | |
|  | | To have a job | | | | |  | | | | | | To live in a country with no war | | | | | | | | | | |  | |
|  | | To have good health | | | | |  | | | | | | To live in a country without violence | | | | | | | | | | |  | |
|  | | To have a good living standard | | | | |  | | | | | | To live in a country without corruption | | | | | | | | | | |  | |
|  | | To get an education (myself) | | | | |  | | | | | | To have a happy family life | | | | | | | | | | |  | |
|  | | To get a good education for my children | | | | |  | | | | | | To live in freedom | | | | | | | | | | |  | |
|  | | To be able to participate in public life | | | | |  | | | | | | To live free of fear | | | | | | | | | | |  | |
|  | | To live a life free of hunger | | | | |  | | | | | | To live free of want | | | | | | | | | | |  | |
|  | | To live independently/ have my own home | | | | |  | | | | | | To live in a country with an effective/good justice system | | | | | | | | | | |  | |
|  | | To be faithful to my religion | | | | |  | | | | | | Other (please specify) | | | | | | | | | | |  | |

**Household Details**

***Next we would like to ask you to answer some questions on your standard of living.***

|  | *****ONLY ASK B1_1 AND B1_2 TO HEAD OF HOUSEHOLD, OTHERWISE GO TO B1_3***** | | | | | | | | | | |
| --- | --- | --- | --- | --- | --- | --- | --- | --- | --- | --- | --- |
| **B1_1** | **Does your household have any of the following? *[read list – circle all that apply]*** | | | | | | | | | | |
|  | Electric iron | | 1 | | Mosquito net/bed net | | | | | 12 | |
|  | Charcoal iron | | 2 | | Mattress | | | | | 13 | |
|  | Refrigerator | | 3 | | Bed | | | | | 14 | |
|  | Deep freezer | | 4 | | Watch or clock | | | | | 15 | |
|  | Television | | 5 | | Sewing machine | | | | | 16 | |
|  | VCR/DVD | | 6 | | Modern Stove | | | | | 17 | |
|  | Radio | | 7 | | Canoe/boat | | | | | 18 | |
|  | Mobile/cell phone | | 8 | | Bicycle | | | | | 19 | |
|  | Computer | | 9 | | Motorcycle | | | | | 20 | |
|  | Generator | | 10 | | Car or truck | | | | | 21 | |
|  | Fan | | 11 | |  | | | | |  | |
| **B1_2** | **What is the main source of drinking water for your household? *[circle one option]*** | | | | | | | | | | |
|  | Piped into residence/compound (go to B1_5) | | 1 | | Rainwater | | | | | 8 | |
|  | Public tap/standpipe | | 2 | | River/stream | | | | | 9 | |
|  | Hand pump in residence/ compound (go to B1_5) | | 3 | | Tanker/truck | | | | | 10 | |
|  | Well in residence/compound (go to B1_5) | | 4 | | Pond/lake | | | | | 11 | |
|  | Bought bottled water | | 5 | | Still water | | | | | 12 | |
|  | Open well | | 6 | | Other (please specify) | | | | | 13 | |
|  | Spring | | 7 | |  |  |  |  |  |  |  |
| **B1_3** | **How long does it take you to walk to the nearest supply of drinking water? *[write in number]*** | | | | | | | | | _______  minutes | |
| **B1_4** | **How safe is it for women, children, persons with disabilities and elders to reach to the nearest supply of drinking water? *[read out the options, circle one option]*** | | | | | | | | | | |
|  | Very safe | a bit safe | | neither safe nor unsafe | | | a bit unsafe | | very unsafe | | |
|  | 1 | 2 | | 3 | | | 4 | | 5 | | |
| *****ONLY ASK B1_5 to B1_7 to HEAD OF HOUSEHOLD. OTHERWISE GO TO B1_8***** | | | | | | | | | | | |
| **B1_5** | **What kind of toilet facility does your household have? *[circle one option]*** | | | | | | | | | | |
|  | Private flush inside | | 1 | | Traditional pit | | | | | 4 | |
|  | Private flush outside | | 2 | | Open defecation near to the house (e.g. beaches or bushes) | | | | | 5 | |
|  | Shared flush (communal) | | 3 | | Other (please specify) | | | | | 6 | |
| **B1_6** | **What is the main cooking fuel *[circle one option]*** | | | | | | | | | | |
|  | Firewood | | 1 | | Crop residue/sawdust | | | | | 6 | |
|  | Charcoal | | 2 | | Animal waste | | | | | 7 | |
|  | Kerosene/oil | | 3 | | Other (please specify) | | | | | 9 | |
|  | Gas | | 4 | | Don’t know | | | | | 88 | |
|  | Electricity | | 5 | | Refused answer | | | | | 99 | |
| **B1_7** | **What is the main source of light the household? *[circle one option]*** | | | | | | | | | | |
|  | Firewood | | 1 | | Solar powered lamp | | | | | 6 | |
|  | Kerosene/oil lamps | | 2 | | Candles | | | | | 7 | |
|  | Gas | | 3 | | Other (please specify) | | | | | 8 | |
|  | Electricity | | 4 | | Don’t know | | | | | 88 | |
|  | Battery powered lamp / light | | 5 | | Refused answer | | | | | 99 | |
| **B1_8** | **How many meals per day do you usually eat? *[write number]*** | | | | | | | _____ | | | |
| **B1_9** | **How many meals did you eat yesterday? *[write number]*** | | | | | | | _____ | | | |
| **B1_10** | **How often do you get enough to eat? *[read out the options, circle one option]*** | | | | | | | | | | |
|  | Always enough | | 1 | | Often not enough | | | | | 3 | |
|  | Often enough | | 2 | | Never enough | | | | | 4 | |
| **B1_11** | **In the last month, did any member of your household have nothing to eat for one or more days?** | | | | | | | | | | |
|  | Yes (go to B1_12) | | 1 | | No/don’t know (go to B1_13) | | | | | | 2 |
|  |  | |  | |  | | | | |  | |
| **B1_12** | **How many days did the member of your household have nothing to eat? *[write number]*** | | | | | ___________ | | | | | |
|  |  | | | | Don’t know how many days | | | | | -88 | |

| *****ONLY ASK B1_13 to B1_17 TO HEAD OF HOUSEHOLD. OTHERWISE GO TO B2_1***** | | | | | | | | | | | | | | | | | |
| --- | --- | --- | --- | --- | --- | --- | --- | --- | --- | --- | --- | --- | --- | --- | --- | --- | --- |
| **What is the condition of the dwelling... *[read out the options, circle one option]*** | | | | | **Very good** | | **a bit good** | | | **a bit poor** | | | | **very poor** | **do not have** | | |
| **B1_13** | | Walls? | | | 1 | | 2 | | | 3 | | | | 4 | 8 | | |
| **B1_14** | | Floors? | | | 1 | | 2 | | | 3 | | | | 4 | 8 | | |
| **B1_15** | | Roof? | | | 1 | | 2 | | | 3 | | | | 4 | 8 | | |
| **B1_16** | | Windows? | | | 1 | | 2 | | | 3 | | | | 4 | 8 | | |
| **B1_17** | | Doors | | | 1 | | 2 | | | 3 | | | | 4 | 8 | | |
| **B2_1** | | | **Not at all satisfied** | | | **a bit unsatisfied** | | | **neither satisfied nor unsatisfied** | | | | **a bit satisfied** | | | **completely satisfied** | |
| How satisfied are you with your own standard of living? ***[read out the options, circle one option]*** | | | 1 | | | 2 | | | 3 | | | | 4 | | | 5 | |
| **B2_2** | | | | **Living with a lot of difficulty** | | | | **Living with a bit of difficulty** | | | **managing** | | **living a bit well** | | | **living very well** | |
| How well do you live based on your current household income? ***[read out the options, circle one option]*** | | | | 1 | | | | 2 | | | 3 | | 4 | | | 5 | |
| **B2_3** | **In the last year has your household living standard… *[read out the options, circle one option]*** | | | | | | | | | | | | | | | | |
|  | Got better | | | | | | | | 1 | | | Don’t know | | | | | 88 |
|  | Stayed the same | | | | | | | | 2 | | | Refused question | | | | | 99 |
|  | Got worse | | | | | | | | 3 | | |  | | | | |  |
| **B2_4** | | | **Poorer** | | | **Fairly poor** | | | **in the middle** | | | | **Fairly rich** | | | **Richer** | |
| How would you compare your standard of living to other households in your community? ***[read out the options, circle one option]*** | | | 1 | | | 2 | | | 3 | | | | 4 | | | 5 | |

**Health And Healthcare Services**

***Next we would like to ask you about your health and access to health services***

| **C1** | **Not at all satisfied** | **a bit unsatisfied** | **neither satisfied or unsatisfied** | **a bit satisfied** | **completely satisfied** |
| --- | --- | --- | --- | --- | --- |
| How satisfied are you with your health overall? ***[read out the options, circle one option]*** | 1 | 2 | 3 | 4 | 5 |

| ***[For C2_1 to C2_7 read out the options, circle one option. If the response is some difficulty, a lot of difficulty or cannot do at all, write the age the respondent started to have difficulty. From birth, write 0]*** | | | | | | | | | **No difficulties** | | **With some difficulty** | **With a lot of difficulty** | | **Cannot do at all** | | **Since when (age)** | | | |  |
| --- | --- | --- | --- | --- | --- | --- | --- | --- | --- | --- | --- | --- | --- | --- | --- | --- | --- | --- | --- | --- |
| **C2_1** | | Do you have difficulty seeing, even if wearing glasses? | | | | | | | 1 | | 2 | 3 | | 4 | | ____ | | | |  |
| **C2_2** | | Do you have difficulty hearing, even if using a hearing aid? | | | | | | | 1 | | 2 | 3 | | 4 | | ____ | | | |  |
| **C2_3** | | Do you have difficulty walking or climbing steps? | | | | | | | 1 | | 2 | 3 | | 4 | | ____ | | | |  |
| **C2_4** | | Do you have difficulty raising a 2 litre bottle of water or soda from waist to eye level? | | | | | | | 1 | | 2 | 3 | | 4 | | ____ | | | |  |
| **C2_5** | | Do you have difficulty remembering or concentrating? | | | | | | | 1 | | 2 | 3 | | 4 | | ____ | | | |  |
| **C2_6** | | Do you have difficulty with self-care such as washing all over or dressing? | | | | | | | 1 | | 2 | 3 | | 4 | | ____ | | | |  |
| **C2_7** | | Do you have difficulty understanding or being understood? | | | | | | | 1 | | 2 | 3 | | 4 | | ____ | | | |  |
| **C2_8** | | **How often do you feel worried, nervous or anxious? *[read out the options, circle one option]*** | | | | | | | | | | | | | | | | | |  |
|  | | Every day | | | | 1 | Never | | | | | | | | | | | 5 | |  |
|  | | Once a week | | | | 2 | Don’t know | | | | | | | | | | | 88 | |  |
|  | | Once a month | | | | 3 | Did not answer | | | | | | | | | | | 99 | |  |
|  | | A few times a year | | | | 4 |  | | | | | | | | | | |  | |  |
| **C2_9** | | **Do you have fits (jerking body movements) or does your body go rigid? *[read out the options, circle one option]*** | | | | | | | | | | | | | | | | | |  |
|  | | Every day | | | | 1 | Never | | | | | | | | | | | 5 | |  |
|  | | Once a week | | | | 2 | Don’t know | | | | | | | | | | | 88 | |  |
|  | | Once a month | | | | 3 | Did not answer | | | | | | | | | | | 99 | |  |
|  | | A few times a year | | | | 4 |  | | | | | | | | | | |  | |  |
| **C3_1** | | **Do you need any devices or support to get around? *[circle one option]*** | | | | | | | | | | | | | | | | | |  |
|  | | Yes (go to C3_2) | | | | 1 | No (go to C4_1) | | | | | | | | | | 2 | | |  |
| **C3_2** | | **What devices or support do you need to get around? *[DO NOT read out the options, circle as many as apply]*** | | | | | | | | | | | | | | | | | |  |
|  | | Walking stick | | | | 1 | Hearing aid | | | | | | | | | | | | 7 |  |
|  | | Crutches | | | | 2 | Glasses | | | | | | | | | | | | 8 |  |
|  | | Wheelchair | | | | 3 | White cane (visual impairment) | | | | | | | | | | | | 9 |  |
|  | | Tricycle | | | | 4 | Communication aids | | | | | | | | | | | | 10 |  |
|  | | Artificial limb* | | | | 5 | Other (please specify) | | | | | | | | | | | | 11 |  |
|  | | Someone’s assistance | | | | 6 |  |  |  |  |  |  |  |  |  |  |  |  |  |  |
| **C3_3** | | **Who helps you move around/mobilise on a regular basis? *[DO NOT read out the options, circle as many as apply]*** | | | | | | | | | | | | | | | | | |  |
|  | | No one | | | | 1 | Other adult male | | | | | | | | | | | | 6 |  |
|  | | Male adult household member | | | | 2 | Other adult female | | | | | | | | | | | | 7 |  |
|  | | Female adult household member | | | | 3 | Other male child | | | | | | | | | | | | 8 |  |
|  | | Male child household member | | | | 4 | Other female child | | | | | | | | | | | | 9 |  |
|  | | Female child household member | | | | 5 |  | | | | | | | | | | | |  |  |
| **C3_4** | | **How often do you have access to these devices or support? *[circle one option]*** | | | | | | | | | | | | | | | | | |  |
|  | | All the time (go to C4_1) | | | | 1 | Occasionally (go to C3_5) | | | | | | | | | | | | 3 |  |
|  | | Most of the time (go to C3_5) | | | | 2 | Never (go to C3_5) | | | | | | | | | | | | 4 |  |
| **C3_5** | | **Why don’t you have the devices you need to get around? *[DO NOT read out the options, circle as many as apply]*** | | | | | | | | | | | | | | | | | |  |
|  | | They cost too much | | | | 1 | They are not available | | | | | | | | | | | | 3 |  |
|  | | I don’t know where to find them | | | | 2 | Other (please specify) | | | | | | | | | | | | 4 |  |
| **C4_1** | | | | **Not at all satisfied** | | **a bit unsatisfied** | | | **neither satisfied or unsatisfied** | | **a bit satisfied** | | | | | **completely satisfied** | | | | |
| How satisfied are you with your access to health services? ***[read out the options, circle one option]*** | | | | 1 | | 2 | | | 3 | | 4 | | | | | 5 | | | | |
| **C4_2** | | **How often can you get the healthcare you need? *[read the options, circle one option]*** | | | | | | | | | | | | | | | | | | |
|  | | All the time | | | 1 | Occasionally/sometimes | | | | | | | | 3 | | | | | | |
|  | | Most of the time | | | 2 | Never | | | | | | | | 4 | | | | | | |
| **C4_3** | | **Where would you go in the case of a health problem or accident? *[DO NOT read out the options, circle as many as apply]*** | | | | | | | | | | | | | | | | | | |
|  | | Private clinic or hospital | | | 1 | Private doctor/dentist | | | | | | | | 8 | | | | | | |
|  | | Government clinic/health centre | | | 2 | Health NGO | | | | | | | | 9 | | | | | | |
|  | | Government hospital | | | 3 | Neighbour | | | | | | | | 10 | | | | | | |
|  | | Pharmacy | | | 4 | Other (please specify)  ____________________ | | | | | | | | 11 | | | | | | |
|  | | Traditional medicine/herbalist | | | 5 | Don’t know | | | | | | | | 88 | | | | | | |
|  | | Mobile clinic/drug peddler | | | 6 | Refused answer | | | | | | | | 99 | | | | | | |
|  | | Religious leader | | | 7 |  | | | | | | | |  | | | | | | |
| **C4_4** | | **Why don’t you have access to healthcare? *[skip if C4_2 = all the time. DO NOT read out the options, circle as many as apply]*** | | | | | | | | | | | | | | | | | | |
|  | | No need | | | 1 | Services are not available/lack of facilities | | | | | | | | 7 | | | | | | |
|  | | It costs too much | | | 2 | Lack of adequate medication/drugs/supplies | | | | | | | | 8 | | | | | | |
|  | | I don’t know where it is | | | 3 | The staff treat me badly/unfriendly staff | | | | | | | | 9 | | | | | | |
|  | | Don’t know the normal opening hours | | | 4 | Long waiting times/takes too long | | | | | | | | 10 | | | | | | |
|  | | The health facility is not accessible to me (no ramps, sign language etc.) | | | 5 | Other (please specify) | | | | | | | | 11 | | | | | | |
|  | | It is too far away/long distance to health facility | | | 6 |  |  |  |  |  |  |  |  |  |  |  |  |  |  |  |
| **C4_5** | | **Do you have problems when you visit the health facility in your community?**  ***[skip if C4_2 = never. DO NOT read out the options, circle as many as apply]*** | | | | | | | | | | | | | | | | | | |
|  | | No problems/satisfied | | | 1 | Treatment unsuccessful | | | | | | | | 10 | | | | | | |
|  | | Facility is not clean | | | 2 | Long distance to health facility | | | | | | | | 11 | | | | | | |
|  | | Long waiting times to see a health service provider | | | 3 | No drugs/medications available (drug stock out, lack of medicine) | | | | | | | | 12 | | | | | | |
|  | | I went to the health facility and they denied me attention | | | 4 | The staff treat me badly/unfriendly staff | | | | | | | | 13 | | | | | | |
|  | | Services not in the local language | | | 5 | The health facility is not accessible to me (no ramps, sign language etc.) | | | | | | | | 14 | | | | | | |
|  | | No ambulance | | | 6 | No female health professionals | | | | | | | | 15 | | | | | | |
|  | | Lack of privacy in examination room | | | 7 | The facility does not offer the correct treatment | | | | | | | | 16 | | | | | | |
|  | | No medical doctor/ trained professionals | | | 8 | Other (please specify) | | | | | | | | 17 | | | | | | |
|  | | I do not have money/too expensive (or high cost of medicine/fees) | | | 9 |  |  |  |  |  |  |  |  |  | | | | | | |

| **C4_6** | **How would you usually get to your health facility? *[circle* ONE *option only*]** | | | | | | | | | | | |
| --- | --- | --- | --- | --- | --- | --- | --- | --- | --- | --- | --- | --- |
|  | Walk | | | | | 1 | Motorbike / Pempem | | | | | 6 |
|  | Government owned bus | | | | | 2 | Tricycle / Keke | | | | |  |
|  | Government ambulance | | | | | 3 | Car taxi | | | | | 7 |
|  | Private car | | | | | 4 | Other (please specify):_______________ | | | | | 8 |
|  | Bicycle | | | | | 5 | Don’t know | | | | | 88 |
| **C4_7** | **Using the above transport, how long does it take to reach your nearest health facility? *[write the number]*** | | | | | | | | | ____________ (minutes) | | |
|  | Don’t know | | | | | -88 | Refused answer | | | | | -99 |
| **C4_8** | **Does the health facility have a private examination room that you can use? *[circle one option]*** | | | | | | | | | | | |
|  | Yes | | | | | 1 | Don’t know | | | | | 88 |
|  | No | | | | | 2 | Refused answer | | | | | 99 |
| **C4_9** | **Does the facility charge user fees for health services? *[circle one option]*** | | | | | | | | | | | |
|  | Yes (go to C4_10) | | | | | 1 | Don’t know (go to C4_11) | | | | | 88 |
|  | No (go to C4_11) | | | | | 2 | Refused answer (go to C4_11) | | | | | 99 |
| **C4_10** | **Which services does the facility charge for? *[read out the options, circle as many as apply]*** | | | | | | | | | | | |
|  | Registration | | | | | 1 | Laboratory Test | | | | | 4 |
|  | Consultation | | | | | 2 | Other (please specify) | | | | | 5 |
|  | Treatment/Drugs | | | | | 3 |  |  |  |  |  |  |
| **C4_11** | **Did you pay for your last health consultation? *[circle one option]*** | | | | | | | | | | | |
|  | Yes | | 1 | | Don't know | | | | | 88 | | |
|  | No | | 2 | | Refused answer | | | | | 99 | | |
| **C4_12** | **How much do you usually spend on your health per month? *[write the number]*** | | | | | | | | _________________ (Liberian $) | | | |
|  | Don’t know | | | | | -88 | Refused answer | | | | | -99 |
| **C4_13** | How satisfied are you with the health care you receive? ***[read out the options, circle one option]*** | **Not at all satisfied** | | **a bit unsatisfied** | | | | **neither satisfied or unsatisfied** | | **a bit satisfied** | **completely satisfied** | |
|  |  | 1 | | 2 | | | | 3 | | 4 | 5 | |

| **C5_1** | | | **Were there any Ebola cases in your community? *[circle one option]*** | | | | | | | | | | | | | | |
| --- | --- | --- | --- | --- | --- | --- | --- | --- | --- | --- | --- | --- | --- | --- | --- | --- | --- |
|  | | | Yes, a few cases | | | 1 | | | | | No Ebola cases | | | | | 3 | |
|  | | | Yes, many cases | | | 2 | | | | | Don’t know | | | | | 88 | |
| **C5_2** | | | **Did the Ebola quarantines affect your household/community? *[circle one option]*** | | | | | | | | | | | | | | |
|  | | | Yes, my household was quarantined | | | 1 | | | | | No | | | | | 4 | |
|  | | | Yes, households in my community were quarantined (not mine) | | | 2 | | | | | Don’t know | | | | | 88 | |
|  | | | Yes, the entire community was quarantined | | | 3 | | | | | Refused answer | | | | | 99 | |
| **C5_3** | | | **How did Ebola affect you and your community? *[DO NOT read out the options, circle as many as apply]*** | | | | | | | | | | | | | | |
|  | | | decreased social life in the community | | | 1 | | | | | people didn't have money for food | | | | | 10 | |
|  | | | stopped communal eating or eating in the same place | | | 2 | | | | | restricted holding of community meetings | | | | | 11 | |
|  | | | stopped welcoming visitors and strangers in the home | | | 3 | | | | | affected traditional culture and practices | | | | | 12 | |
|  | | | restricted movement of persons in and out of the community | | | 4 | | | | | changed the way people took care of the sick in the family and community | | | | | 13 | |
|  | | | many orphaned children | | | 5 | | | | | Loss of job/livelihood | | | | | 14 | |
|  | | | community members did not seek health treatment at health facilities for fear of being isolated or rejected | | | 6 | | | | | negative treatment and stigma of Ebola affected people (e.g. orphans, survivors and relatives) | | | | | 15 | |
|  | | | close down of health facilities | | | 7 | | | | | Don’t know | | | | | 88 | |
|  | | | close down of schools | | | 8 | | | | | Refused answer | | | | | 99 | |
|  | | | people didn't have enough money to send children back to school | | | 9 | | | | | Other (please specify) | | | | | 16 | |
| **C5_4** | | | **Who did you and your community listen to when making decisions about how to stop the spread of Ebola? *[DO NOT read out the options, circle as many as apply]*** | | | | | | | | | | | | | | |
|  | | | community leaders | | | 1 | | | | | Family members | | | | | 7 | |
|  | | | religious leaders | | | 2 | | | | | Friends/neighbours | | | | | 8 | |
|  | | | traditional leaders | | | 3 | | | | | Government/local authority representatives | | | | | 9 | |
|  | | | disabled people’s organizations (DPOs) | | | 4 | | | | | Other (please specify) _________________ | | | | | 10 | |
|  | | | Teachers/school administrators | | | 5 | | | | | Don’t know | | | | | 88 | |
|  | | | health workers | | | 6 | | | | |  | | | | |  | |
| **C5_5** | | | **What were the main problems you and your community faced during the Ebola outbreak? *[DO NOT read out the options, circle as many as apply]*** | | | | | | | | | | | | | | |
|  | | | lack of information on the Ebola outbreak | | | 1 | | | | | worse sanitation | | | | | 10 | |
|  | | | lack of information on types of health services available in health facilities | | | 2 | | | | | lack of access to medical care/essential drugs/vaccines | | | | | 11 | |
|  | | | Closure of health facilities | | | 3 | | | | | Children out of school | | | | | 12 | |
|  | | | lack of access to Ebola Treatment Units (ETU) | | | 4 | | | | | Loss of job/livelihood | | | | | 13 | |
|  | | | lack of food | | | 5 | | | | | Unfriendly attitude of health workers | | | | | 14 | |
|  | | | lack of safe drinking water | | | 6 | | | | | not able to bury loved ones in the usual way | | | | | 15 | |
|  | | | lack of access to water, soap and chlorine | | | 7 | | | | | People afraid of going to health facilities fearing exposure to Ebola | | | | | 16 | |
|  | | | Loss of freedom during quarantine | | | 8 | | | | | Negative perception of quarantine as shameful | | | | | 17 | |
|  | | | lack of information on accessible services for vulnerable groups (e.g. persons with HIV/AIDS and/or TB or persons with disabilities) | | | 9 | | | | | Other (please specify) | | | | | 18 | |
| **C5_6** | | | **In your opinion, who suffered the most during the Ebola outbreak? *[read out ALL the options, circle ONE option]*** | | | | | | | | | | | | | | |
|  | | | orphaned children whose parents died because of Ebola | | | 1 | | | | | Community health volunteers | | | | | 9 | |
|  | | | relatives of people who died because of Ebola | | | 2 | | | | | Persons with disabilities | | | | | 10 | |
|  | | | survivors of Ebola | | | 3 | | | | | Persons with HIV/AIDS or TB | | | | | 11 | |
|  | | | relatives of survivors of Ebola | | | 4 | | | | | Older people | | | | | 12 | |
|  | | | Health workers | | | 5 | | | | | Religious leaders | | | | | 13 | |
|  | | | Ambulance teams | | | 6 | | | | | Pregnant Women | | | | | 14 | |
|  | | | Traditional healers | | | 7 | | | | | Other (please specify) | | | | | 15 | |
|  | | | Burial teams | | | 8 | | | | | Don’t know | | | | | 88 | |
| **C5_7** | | **During the Ebola outbreak, did your access to health services *[read out the options, circle one option]*** | | | | | | | | | | | | | | | |
|  | | Get better | | | | | | 1 | | | | Get worse | | | 3 | | |
|  | | Stay the same | | | | | | 2 | | | | Don’t know | | | 88 | | |
| **C5_8** | | **How did you get treatment during the Ebola outbreak?** | | | | | | | | | | | | | | | |
|  | | Did not need treatment | | | | | | 1 | | | | Other (please specify) | | | 5 | | |
|  | | Health facilities | | | | | | 2 | | | |  |  |  |  |  |  |
|  | | Traditional healers | | | | | | 3 | | | | Don’t know | | | 88 | | |
|  | | Self-treatment/treatment from household members | | | | | | 4 | | | | Refused answer | | | 99 | | |
| **C5_9** | | | **During the Ebola outbreak, did your community change how they acted towards anyone/any groups of people?** | | | | | | | | | | | | | | |
|  | | | Yes (go to C5_10) | | | | | | 1 | No/don’t know (go to C6_1) | | | | | | | 2 |
| **C5_10** | | | **Which groups/people were treated differently? *[read out ALL the options, circle as many as apply]*** | | | | | | | | | | | | | | |
|  | | | orphaned children whose parents died because of Ebola | | | | | | 1 | Community health volunteers | | | | | | | 8 |
|  | | | relatives of people who died because of Ebola | | | | | | 2 | Persons with disabilities | | | | | | | 9 |
|  | | | survivors of Ebola | | | | | | 3 | Persons with HIV/AIDS or TB | | | | | | | 10 |
|  | | | relatives of survivors of Ebola | | | | | | 4 | Older people | | | | | | | 11 |
|  | | | Health workers | | | | | | 5 | Religious leaders | | | | | | | 12 |
|  | | | Ambulance teams | | | | | | 6 | Other (please specify) | | | | | | | 13 |
|  | | | Burial teams | | | | | | 7 | Don’t know | | | | | | | 88 |
| **C5_11** | | | **How were they treated differently? *[DO NOT read the options, circle as many as apply]*** | | | | | | | | | | | | | | |
|  | | | they were not allowed to return home | | | | | | 1 | they were refused transportation in “collective” car taxis and “pen-pen” motorbikes | | | | | | | 6 |
|  | | | they were isolated in the community | | | | | | 2 | they were stopped from returning to their regular jobs | | | | | | | 7 |
|  | | | they were treated as an outsider | | | | | | 3 | the issue of being a survivor became a taboo | | | | | | | 8 |
|  | | | they were rejected and shunned by others or treated as inferior | | | | | | 4 | Other (please specify) | | | | | | | 9 |
|  | | | they were not treated fairly | | | | | | 5 | Don’t know | | | | | | | 88 |
| ***[only ask C5_12 and C5_13 if the respondent is a person with disabilities, otherwise go to C6_1]*** | | | | | | | | | | | | | | | | | |
| **C5_12** | **During the Ebola outbreak, did people change how they acted towards you? *[circle one option]*** | | | | | | | | | | | | | | | | |
|  | Yes (go to C5_13) | | | | | | 1 | | | Don’t know (go to C6_1) | | | | | | 88 | |
|  | No (go to C6_1) | | | | | | 2 | | | Refused question (go to C6_1) | | | | | | 99 | |
| **C5_13** | **How did the way they act change? *[DO NOT read the options, circle as many as apply]*** | | | | | | | | | | | | | | | | |
|  | I was not allowed to return home | | | | | | 1 | | | I was refused transportation in “collective” car taxis and “pen-pen” motorbikes | | | | | | 6 | |
|  | I felt isolated in the community | | | | | | 2 | | | I was stopped from returning to my regular jobs | | | | | | 7 | |
|  | I was treated as an outsider | | | | | | 3 | | | the issue of being a survivor became a taboo | | | | | | 8 | |
|  | I felt rejected and shunned by others or treated as inferior | | | | | | 4 | | | Other (please specify) __________________ | | | | | | 9 | |
|  | I was not treated fairly | | | | | | 5 | | | Don’t know | | | | | | 88 | |
| **C6_1** | | | | **Compared to your life before the Ebola is your life… *[read out the options, circle one option]*** | | | | | | | | | | | | | |
|  | | | | Much better | A bit better | No change | | | | | | | A bit worse | Much worse | | | |
|  | | | | 1 | 2 | 3 | | | | | | | 4 | 5 | | | |
| **C6_2** | | | | **Compared to your life before the Ebola outbreak do you have… *[read out the options, circle one option]*** | | | | | | | | | | | | | |
|  | | | | Much more money | A bit more money | No change | | | | | | | A bit less money | Much less money | | | |
|  | | | | 1 | 2 | 3 | | | | | | | 4 | 5 | | | |
| **C6_3** | | | | **Compared to your life before the Ebola outbreak are you… *[read out the options, circle one option]*** | | | | | | | | | | | | | |
|  | | | | Much more happy | A bit more happy | No change | | | | | | | A bit more unhappy | Much more unhappy | | | |
|  | | | | 1 | 2 | 3 | | | | | | | 4 | 5 | | | |

**Education**

***Now I would like to ask you some questions about school and education***

| **D1** | | **What is the highest level of education you have completed? *[circle one option]*** | | | | | | | | | | | | |  |
| --- | --- | --- | --- | --- | --- | --- | --- | --- | --- | --- | --- | --- | --- | --- | --- |
|  | | No formal education (go to D2_1) | | | | 1 | | Some College (go to D3_1) | | | | | 6 | |  |
|  | | Some primary (go to D2_1) | | | | 2 | | Completed College (go to D3_1)) | | | | | 7 | |  |
|  | | Completed primary (go to D2_1) | | | | 3 | | Some university (go to D3_1) | | | | | 8 | |  |
|  | | Some secondary (go to D2_1) | | | | 4 | | University (go to D3_1) | | | | | 9 | |  |
|  | | Completed secondary (go to D3_1) | | | | 5 | | Other (specify) (go to D2_1) | | | | | 10 | |  |
| ***[for D2_1 and D2_2 read the options, circle one option]*** | | | | | | | Well | | | With some  difficulty | With a lot of difficulty | Cannot do at all | | |  |
| **D2_1** | | | Can you read and write? | | | | 1 | | | 2 | 3 | 4 | | |  |
| **D2_2** | | | Can you do maths/sums? | | | | 1 | | | 2 | 3 | 4 | | |  |
| **D3_1** | | How satisfied are you with the education/ school in your community?  ***[read the options, circle one option]*** | | | **Not at all satisfied** | **a bit unsatisfied** | | | | **neither satisfied or unsatisfied** | | **a bit satisfied** | **completely Satisfied** | | **Don't know** |
|  |  |  |  |  | 1 | 2 | | | | 3 | | 4 | 5 | | 88 |
| **D3_2** | | How important do you think it is for a child to go to school?  ***[read the options, circle one option]*** | | | **Very unimportant** | **a bit unimportant** | | | | **neither important or unimportant** | | **a bit important** | **very important** | | **Don't know** |
|  |  |  |  |  | 1 | 2 | | | | 3 | | 4 | 5 | | 88 |
| **D3_3** | | How important is education for persons with disabilities?  ***[read the options, circle one option]*** | | | **Very unimportant** | **a bit unimportant** | | | | **neither important or unimportant** | | **a bit important** | **very important** | | **Don't know** |
|  |  |  |  |  | 1 | 2 | | | | 3 | | 4 | 5 | | 88 |

**Work and Employment**

***Now I would like to ask you some questions about work and employment. By work and employment we mean a job where you get paid, your own business or income from farming.***

| **E1_1** | | **What is your main source of income? *[DO NOT read out the options, circle one option]*** | | | | | | | | | | | | | | | | | | | |
| --- | --- | --- | --- | --- | --- | --- | --- | --- | --- | --- | --- | --- | --- | --- | --- | --- | --- | --- | --- | --- | --- |
|  | | No income | | | | | | | | 1 | | Money sent home from abroad | | | | | | | | | 7 |
|  | | Formal paid employment | | | | | | | | 2 | | Money sent home from within Liberia | | | | | | | | | 8 |
|  | | Self-employed | | | | | | | | 3 | | Street begging | | | | | | | | | 9 |
|  | | Family support/donation | | | | | | | | 4 | | Government aid | | | | | | | | | 10 |
|  | | Other individual support (not from family/relatives) | | | | | | | | 5 | | Don't know | | | | | | | | | 88 |
|  | | Aid from international/civil society organisation | | | | | | | | 6 | | Other (please specify) | | | | | | | | | 11 |
| **E1_2** | | **Do you receive any financial or welfare assistance?** | | | | | | | | | | | | | | | | | | | |
|  | | Yes (go to E1_3) | | | | | | | | 1 | | Don’t know (go to E1_4) | | | | | | | | | 88 |
|  | | No (go to E1_4) | | | | | | | | 2 | | Refused answer (go to E1_4) | | | | | | | | | 99 |
| **E1_3** | | **What kind of assistance do you receive?** ***[DO NOT read out the options, circle as many as apply]*** | | | | | | | | | | | | | | | | | | | |
|  | | National pension scheme | | | | | | | | 1 | | Social cash transfer | | | | | | | | | 5 |
|  | | Employment injury scheme | | | | | | | | 2 | | School take-home rations | | | | | | | | | 6 |
|  | | Survivor’s pension | | | | | | | | 3 | | Other (please specify) | | | | | | | | | 7 |
|  | | Refund | | | | | | | | 4 | |  |  |  |  |  |  |  |  |  |  |
| **E1_4** | | **How much money do you make? *[fill in the number, circle week or month]*** | | | | | | | | | | | | | __________ (Liberian $)  Per week / per month | | | | | | |
|  | | Don’t know | | | -88 | | | | Refused answer | | | | | | | | | -99 | | | |
|  | | *****ONLY ASK E1_5 to HEAD OF HOUSEHOLD. OTHERWISE GO TO E1_6***** | | | | | | | | | | | | | | | | | | | |
| **E1_5** | | **How many people in your household currently have a job where they get paid or have their own business or get income from farming? *[write the number]*** | | | | | | | | | | | | |  | | | | | | |
| **E1_6** | | **Do you have a job where you get paid or your own business or income from farming?** | | | | | | | | | | | | | | | | | | | |
|  | | Yes. (go to E1_10) | | | | | | | | 1 | | No (go to E1_7) | | | | | | | | | 2 |
| **E1_7** | | **Are you looking for a paid job or to start your own business or to get income from farming?** | | | | | | | | | | | | | | | | | | | |
|  | | Yes (go to E1_8) | | | | | | | | 1 | | No (go to E1_9) | | | | | | | | | 3 |
| **E1_8** | | **Where are you looking to find work or employment? (then go to E1_10) *[DO NOT read out the options, circle as many as apply]*** | | | | | | | | | | | | | | | | | | | |
|  | | Asking members of my family | | | | | | | | 1 | | Asking for money from banks/micro-finance to start an activity | | | | | | | | | 5 |
|  | | Asking friends/neighbours | | | | | | | | 2 | | Checking advertisements in the newspaper/internet | | | | | | | | | 6 |
|  | | Asking people I meet in the street/going door-to-door | | | | | | | | 3 | | Nothing, I am waiting for someone to offer me a job | | | | | | | | | 7 |
|  | | Asking for money from family/friends to start an activity | | | | | | | | 4 | | Other (please specify) | | | | | | | | | 8 |
| **E1_9** | | **Why are you not looking for work or employment? (then go to E1_12) *[DO NOT read out the options, circle as many as apply]*** | | | | | | | | | | | | | | | | | | | |
|  | | I have found a job that I will start soon | | | | | | | | 1 | | I don’t have the right skills | | | | | | | | | 5 |
|  | | There are no job opportunities | | | | | | | | 2 | | No one is pushing me to find a job | | | | | | | | | 6 |
|  | | No one offered me a job | | | | | | | | 3 | | I don’t really need a job | | | | | | | | | 7 |
|  | | No one wanted to hire a disabled person | | | | | | | | 4 | | I have health problems | | | | | | | | | 8 |
|  | |  | | | | | | | |  | | Other (please specify) __________________ | | | | | | | | | 9 |
| **E1_10** | | **What kind of work or employment do you have? *[DO NOT read out the options, circle as many as apply]*** | | | | | | | | | | | | | | | | | | | |
|  | | Employed in a fixed job | | | | | | | | 1 | | I am a contributing family worker | | | | | | | | | 5 |
|  | | Employed in occasional work | | | | | | | | 2 | | Apprentice | | | | | | | | | 6 |
|  | | Helping someone with his/her work | | | | | | | | 3 | | I have my own business and I have employees | | | | | | | | | 7 |
|  | | Self-employed | | | | | | | | 4 | | Other (please specify) | | | | | | | | | 8 |
| **E1_11** | | How satisfied are you with your work/ employment?  ***[read the options, circle one option]*** | | **Not at all satisfied** | | | | **a bit unsatisfied** | | | | | | **neither satisfied or unsatisfied** | | | **a bit satisfied** | | **completely satisfied** | | |
|  |  |  |  | 1 | | | | 2 | | | | | | 3 | | | 4 | | 5 | | |
| ****ONLY ASK E1_12 to E1_15 TO THE HEAD OF HOUSEHOLD. OTHERWISE GO TO E2_1***** | | | | | | | | | | | | | | | | | | | | | |
| **E1_12** | | **What is the household’s main source of income? *[DO NOT read the options, circle one option]*** | | | | | | | | | | | | | | | | | | | |
|  | | No income | | | | | | | | 1 | | Money sent home from abroad | | | | | | | | | 7 |
|  | | Formal paid employment | | | | | | | | 2 | | Money sent home from within Liberia | | | | | | | | | 8 |
|  | | Self-employed | | | | | | | | 3 | | Street begging | | | | | | | | | 9 |
|  | | Family support/donation | | | | | | | | 4 | | Government aid | | | | | | | | | 10 |
|  | | Other individual support (not from family/relatives) | | | | | | | | 5 | | Don't know | | | | | | | | | 88 |
|  | | Aid from international/civil society organisation | | | | | | | | 6 | | Other (please specify) | | | | | | | | | 11 |
| **E1_13** | How stable is your household income?  ***[read the options, circle one option]*** | | **Not at all stable** | | | | **A bit unstable** | | | | | | **Neither stable nor unstable** | | | **A bit stable** | | | | **Completely stable** | |
|  |  |  | 1 | | | | 2 | | | | | | 3 | | | 4 | | | | 5 | |
|  |  |  |  | | | | | | | | | | | | | Don’t know | | | | 88 | |
| **E1_14** | **What is the monthly rent? *[write in number]*** | | | | | | | | | | (Liberian $) | | | | | | | | | | |
| **E1_15** | **What is the weekly expenditure of the household? *[write in each number]*** | | | | | | | | | | | | | | | | | | | | |
|  | Food | | | | | | | | | L$ | |  | | | | | | | | | -88 |
|  | Communication | | | | | | | | | L$ | |  | | | | | | | | | -88 |
|  | Transport | | | | | | | | | L$ | |  | | | | | | | | | -88 |
|  | Other expenditure | | | | | | | | | L$ | |  | | | | | | | | | -88 |
|  | **TOTAL** | | | | | | | | | **L$** | |  | | | | | | | | | -88 |
|  | Don’t know | | | | | | | | | -88 | | Refused answer | | | | | | | | | -99 |
| **E2_1** | **Do you do any chores in the house? *[DO NOT read out the options, circle as many as apply]*** | | | | | | | | | | | | | | | | | | | | |
|  | No chores | | | | | | | | | 1 | | Taking care of persons with disabilities in the family | | | | | | | | | 7 |
|  | Cooking | | | | | | | | | 2 | | Laundry/washing clothes | | | | | | | | | 8 |
|  | Cleaning | | | | | | | | | 3 | | Groceries/everyday shopping | | | | | | | | | 9 |
|  | Drawing/fetching water | | | | | | | | | 4 | | Cutting wood | | | | | | | | | 10 |
|  | Taking care of elderly family members | | | | | | | | | 5 | | Other (please specify)  ______________________ | | | | | | | | | 11 |
|  | Taking care of children in the family | | | | | | | | | 6 | |  |  |  |  |  |  |  |  |  |  |
| **E2_2** | **How many hours per week do you work/do chores? *[write in number, skip if no work AND no chores]*** | | | | | | | | | | | | | | | | | | | | |
|  | Work | | | | | (hours per week) | | | | | | | | | | | | | | | |
|  | Chores | | | | | (hours per week) | | | | | | | | | | | | | | | |
| **E2_3** | **Does anyone else do chores around the house *[read out the options, circle as many as apply]*** | | | | | | | | | | | | | | | | | | | | |
|  | No | | | | | | | | | 1 | | Niece/nephew | | | | | | | | | 6 |
|  | Parent/grandparent | | | | | | | | | 2 | | Grandchild | | | | | | | | | 7 |
|  | Husband/wife/partner | | | | | | | | | 3 | | Non-family household member | | | | | | | | | 8 |
|  | Son/daughter | | | | | | | | | 4 | | Household employee | | | | | | | | | 9 |
|  | Brother/sister | | | | | | | | | 5 | | Other (please specify) __________________ | | | | | | | | | 10 |
|  |  | | | | | | | | |  | |  |  |  |  |  |  |  |  |  |  |

**Transport**

**We would now like to ask you some questions about access to transport**

| **F1_1** | | | How satisfied are you with the access to transport in your community? ***[read the options, circle one option]*** | **Not at all satisfied** | **A bit unsatisfied** | | | | | **neither satisfied or unsatisfied** | **A bit satisfied** | **completely satisfied** | | |
| --- | --- | --- | --- | --- | --- | --- | --- | --- | --- | --- | --- | --- | --- | --- |
|  |  |  |  | 1 | 2 | | | | | 3 | 4 | 5 | | |
| **F1_2** | | **What kind of transport do you have access to? *[read out the options, circle as many as apply]*** | | | | | | | | | | | | |
|  | | Own car | | | | 1 | | | Own motorbike | | | | | 7 |
|  | | Someone else’s car | | | | 2 | | | Bike | | | | | 8 |
|  | | Motorbike taxi (Pempem) | | | | 3 | | | Walking | | | | | 9 |
|  | | Tricycle / Keke | | | | 4 | | | Other (please specify) | | | | | 10 |
|  | | Car taxi (collective) | | | | 5 | | | Don’t know | | | | | 88 |
|  | | Bus | | | | 6 | | |  | | | | |  |
| **F1_3** | **How often do you have access to the transport you need? *[read the options, circle one option]*** | | | | | | | | | | | | | |
|  | Never (go to F1_4) | | | | | | 1 | Most of the time (go to F1_4) | | | | | 3 | |
|  | Sometimes (go to F1_4) | | | | | | 2 | All of the time (go to G1_1) | | | | | 4 | |
| **F1_4** | **Why don’t you have access? *[DO NOT read out the options, circle as many as apply]*** | | | | | | | | | | | | | |
|  | Not available | | | | 1 | | | Not safe | | | | | 5 | |
|  | I don't want to use it | | | | 2 | | | Don’t know | | | | | 88 | |
|  | Costs too much | | | | 3 | | | Other (please specify) _____________________ | | | | | 6 | |
|  | Not disability accessible | | | | 4 | | |  | | | | |  | |

**Social interactions**

Now I would like to ask you some questions about social interactions

| **G1_1** | | How included do you feel in your community? ***[read the options, circle one option]*** | | | | | | **Very included** | | **A bit included** | | | | | | | | | **neither included nor not included** | | | | | | | **A bit not included** | | | | **Not included at all** | | | | |
| --- | --- | --- | --- | --- | --- | --- | --- | --- | --- | --- | --- | --- | --- | --- | --- | --- | --- | --- | --- | --- | --- | --- | --- | --- | --- | --- | --- | --- | --- | --- | --- | --- | --- | --- |
|  |  |  |  |  |  |  |  | 1 | | 2 | | | | | | | | | 3 | | | | | | | 4 | | | | 5 | | | | |
| **G1_2** | | **Are you a member of any groups or associations? *[DO NOT read out the options, circle as many as apply]*** | | | | | | | | | | | | | | | | | | | | | | | | | | | | | | | | |
|  | | No group | | | | | | | | | | | | | | | 0 | | Parent’s and teachers’ association (PTA) | | | | | | | | | | | | | | | 8 |
|  | | Religious institution/group (church or mosque) | | | | | | | | | | | | | | | 1 | | Other community based/civil society organization | | | | | | | | | | | | | | | 9 |
|  | | Youth/student’s association | | | | | | | | | | | | | | | 2 | | Market association | | | | | | | | | | | | | | | 10 |
|  | | Women’s association | | | | | | | | | | | | | | | 3 | | Trade union | | | | | | | | | | | | | | | 11 |
|  | | Men’s association | | | | | | | | | | | | | | | 4 | | Other professional association | | | | | | | | | | | | | | | 12 |
|  | | Disabled people's organisation (DPO) | | | | | | | | | | | | | | | 5 | | Political party | | | | | | | | | | | | | | | 13 |
|  | | Human rights organisation | | | | | | | | | | | | | | | 6 | | Other (please specify) | | | | | | | | | | | | | | | 14 |
|  | | Traditional society | | | | | | | | | | | | | | | 7 | |  |  |  |  |  |  |  |  |  |  |  |  |  |  |  |  |
| **G1_3** | | **Do you participate in any community activities?** | | | | | | | | | | | | | | | | | | | | | | | | | | | | | | | | |
|  | | Yes (go to G1_4) | | | | | | | | | | | | | | | 1 | | No (go to G1_5) | | | | | | | | | | | | | | | 2 |
| **G1_4** | **What kinds of community activities? (then go to G1_6) *[read out the options, circle as many as apply]*** | | | | | | | | | | | | | | | | | | | | | | | | | | | | | | | | | |
|  | Petty trading | | | | | | | | | | 1 | | | | | | | Hair dressing | | | | | | | | | | | | | | 6 | | |
|  | Music | | | | | | | | | | 2 | | | | | | | Social clubs | | | | | | | | | | | | | | 7 | | |
|  | Fishing | | | | | | | | | | 3 | | | | | | | Sports | | | | | | | | | | | | | | 8 | | |
|  | Community farming | | | | | | | | | | 4 | | | | | | | Religious activities | | | | | | | | | | | | | | 9 | | |
|  | Community loan/savings club | | | | | | | | | | 5 | | | | | | | Other (please specify) | | | | | | | | | | | | | | 10 | | |
| **G1_5** | **Why don’t you participate in community activities? *[DO NOT read out the options, circle as many as apply]*** | | | | | | | | | | | | | | | | | | | | | | | | | | | | | | | | | |
|  | I do not wish to participate | | | | | | | | 1 | | | | | | | I was rejected because a member of my household has a disability | | | | | | | | | | | | | | | | | 6 | |
|  | It costs too much money | | | | | | | | 2 | | | | | | | I cannot get around, lack of transport/ inaccessible transport | | | | | | | | | | | | | | | | | 7 | |
|  | I think I would be rejected | | | | | | | | 3 | | | | | | | The premises are not disability accessible | | | | | | | | | | | | | | | | | 8 | |
|  | I am afraid to do so | | | | | | | | 4 | | | | | | | The attitude of other community members put me off | | | | | | | | | | | | | | | | | 9 | |
|  | I tried but I was rejected because of my disability | | | | | | | | 5 | | | | | | | Other (please specify) | | | | | | | | | | | | | | | | | 10 | |
| **G1_6** | **Do you have friends? *[read out the options, circle one option]*** | | | | | | | | | | | | | | | | | | | | | | | | | | | | | | | | | |
|  | Yes, many/enough (go to G2_1) | | | | | | | | | | | | | | | | | 1 | | | | | No (go to G2_2) | | | | | | | | | 3 | | |
|  | Yes, a few/not enough (go to G2_1) | | | | | | | | | | | | | | | | | 2 | | | | |  | | | | | | | | |  | | |
| **G2_1** | | | How satisfied are you with your relationships with friends | | | | **Not at all satisfied** | | | | | | | **A bit unsatisfied** | | | | | | | **neither satisfied or unsatisfied** | | | | **A bit satisfied** | | | | **completely satisfied** | | | | | |
|  |  |  |  |  |  |  | 1 | | | | | | | 2 | | | | | | | 3 | | | | 4 | | | | 5 | | | | | |
| **G2_2** | | | How satisfied are you with your relationships with your household? | | | | **Not at all satisfied** | | | | | | | **A bit unsatisfied** | | | | | | | **neither satisfied or unsatisfied** | | | | **A bit satisfied** | | | | **completely satisfied** | | | | | |
|  |  |  |  |  |  |  | 1 | | | | | | | 2 | | | | | | | 3 | | | | 4 | | | | 5 | | | | | |
|  | | | ***[only ask if the respondent is in a personal relationship – check question A5_2]*** | | | | | | | | | | | | | | | | | | | | | | | | | | | | | | | |
| **G2_3** | | | How satisfied are you with your relationship with your husband/ wife/partner | | | **Not at all satisfied** | | | | | | **A bit unsatisfied** | | | | | | | | | **neither satisfied or unsatisfied** | | | | **A bit satisfied** | | | | **completely satisfied** | | | | | |
|  |  |  |  |  |  | 1 | | | | | | 2 | | | | | | | | | 3 | | | | 4 | | | | 5 | | | | | |
|  | | | | ***[G3_1 to G3_4 read the options, circle one option]*** | | | | | | | | | | | **All the time** | | | | | | | **Most of the time** | | | | | **Not often** | | | | **Never** | | | |
| **G3_1** | | | | Do your neighbours help you when you ask for assistance | | | | | | | | | | | 1 | | | | | | | 2 | | | | | 3 | | | | 4 | | | |
| **G3_2** | | | | Do you help your neighbours when they ask for assistance | | | | | | | | | | | 1 | | | | | | | 2 | | | | | 3 | | | | 4 | | | |
| **G3_3** | | | | Does your community help when you ask for assistance? | | | | | | | | | | | 1 | | | | | | | 2 | | | | | 3 | | | | 4 | | | |
| **G3_4** | | | | Do you help your community in community initiatives? | | | | | | | | | | | 1 | | | | | | | 2 | | | | | 3 | | | | 4 | | | |
| **G3_5** | How much do you trust your neighbours? | | | | **Not at all** | | | | | | | | **not very much** | | | | | | | **no opinion** | | | | **a bit** | | | | **completely** | | | | | | |
|  |  |  |  |  | 1 | | | | | | | | 2 | | | | | | | 3 | | | | 4 | | | | 5 | | | | | | |
| **G4_1** | | **Do you vote? *[read out the options, circle one option]*** | | | | | | | | | | | | | | | | | | | | | | | | | | | | | | | | |
|  | | Yes, always (go to G4_3) | | | | | | | | | | | | | | | 1 | | No (go to G4_2) | | | | | | | | | | | | | | | 3 |
|  | | Yes, sometimes (go to G4_2) | | | | | | | | | | | | | | | 2 | | Refused question (go to G4_3) | | | | | | | | | | | | | | | 99 |
| **G4_2** | **Why don’t you vote/always vote? *[DON’T read out the options, circle as many as apply]*** | | | | | | | | | | | | | | | | | | | | | | | | | | | | | | | | | |
|  | I am not registered to vote | | | | | | | | 1 | | | | | | | | | The polling station is not accessible to me | | | | | | | | | | | | | | 5 | | |
|  | I don’t think it will make a difference | | | | | | | | 2 | | | | | | | | | I tried to vote but I was turned away | | | | | | | | | | | | | | 6 | | |
|  | I can’t get to the polling station | | | | | | | | 3 | | | | | | | | | Other (please specify) | | | | | | | | | | | | | | 7 | | |
|  | I can’t read the ballot paper | | | | | | | | 4 | | | | | | | | | Don’t know | | | | | | | | | | | | | | 88 | | |
| **G4_3** | | How included do you feel in the decision making of your community? ***[read the options, circle one option]*** | | | | | | **Very included** | | **A bit included** | | | | | | | | | **neither included nor not included** | | | | | | | **A bit not included** | | | | **Not included at all** | | | | |
|  |  |  |  |  |  |  |  | 1 | | 2 | | | | | | | | | 3 | | | | | | | 4 | | | | 5 | | | | |

**Crime and safety**

***We would now like to ask you some questions about crime and safety***

| **H1_1** | **In the last year has the level of crime in your community… *[read out the options, circle one option]*** | | | | |
| --- | --- | --- | --- | --- | --- |
|  | Got better | 1 | | Got worse | 3 |
|  | Stayed the same | 2 | | Don't know | 88 |
| **H1_2** | **In the last year has your community been affected by any conflict or dispute?** | | | | |
|  | Yes | 1 | | Don't know | 88 |
|  | No | 2 | | Refused answer | 99 |
| **H1_3** | **Have you personally experienced any form of crime or violence in the last year? *[read out the options, circle one option]*** | | | | |
|  | Yes, more than once (go to H1_4) | 1 | Never experienced crime or violence (go to H1_5) | | 4 |
|  | Yes, once (go to H1_4) | 2 | Don’t know (go to H1_5) | | 88 |
|  | Not in the last year (go to H1_5) | 3 | Did not answer (go to H1_5) | | 99 |
| **H1_4** | **What sort of crime or violence? *[DO NOT read out the options, circle as many as apply]*** | | | | |
|  | Assault, beating or physical injury | 1 | Child abuse or neglect | | 8 |
|  | Theft or robbery | 2 | Child trafficking, child sexual exploitation or child prostitution | | 9 |
|  | Sexual abuse/harassment or rape/attempted rape | 3 | Eviction from the household/community | | 10 |
|  | Sexual exploitation or prostitution | 4 | Forced labour | | 11 |
|  | Witness murder | 5 | Don't know/not sure | | 88 |
|  | Intimidation or death threats | 6 | Refused answer | | 99 |
|  | Ethnic/racial violence or mob violence | 7 | Other (please specify) | | 12 |
| **H1_5** | **Has anyone in your household witnessed any crime or violence in the last year? *[read out the options, circle one option]*** | | | | |
|  | Yes, more than once (go to H1_6) | 1 | Never witnessed crime or violence (go to H2_1) | | 4 |
|  | Yes, once (go to H1_6) | 2 | Don’t know (go to H2_1) | | 88 |
|  | Not in the last year (go to H2_1) | 3 | Did not answer (go to H2_1) | | 99 |
| **H1_6** | **What sort of crime or violence? *[DO NOT read out the options, circle as many as apply]*** | | | | |
|  | Assault, beating or physical injury | 1 | Child abuse or neglect | | 8 |
|  | Theft or robbery | 2 | Child trafficking, child sexual exploitation or child prostitution | | 9 |
|  | Sexual abuse/harassment or rape/attempted rape | 3 | Eviction from the household/community | | 10 |
|  | Sexual exploitation or prostitution | 4 | Forced labour | | 11 |
|  | Witness murder | 5 | Don't know/not sure | | 88 |
|  | Intimidation or death threats | 6 | Refused answer | | 99 |
|  | Ethnic/racial violence or mob violence | 7 | Other (please specify) | | 12 |

| **H2_1** | | | **How often do you feel safe in your home? *[read out the options, circle one option]*** | | | | | | | | | | | | | |  |
| --- | --- | --- | --- | --- | --- | --- | --- | --- | --- | --- | --- | --- | --- | --- | --- | --- | --- |
|  | | | Never | | | | | | 1 | | Most of the time | | | | 3 | |  |
|  | | | Sometimes | | | | | | 2 | | All of the time | | | | 4 | |  |
| **H2_2** | | | **How often do you feel safe outside of your home? *[read out the options, circle one option]*** | | | | | | | | | | | | | |  |
|  | | | Never | | | | | | 1 | | Most of the time | | | | 3 | |  |
|  | | | Sometimes | | | | | | 2 | | All of the time | | | | 4 | |  |
| **H3_1** | | | **Do you know where to go if you are victim of crime?** | | | | | | | | | | | | | | |
|  | | | Yes (go to H3_2) | | | | | 1 | | | No/not sure (go to H4_1) | | | | | | 2 |
| **H3_2** | | | **Where would you go if you were a victim of crime? *[DO NOT read out the options, circle as many as apply]*** | | | | | | | | | | | | | | |
|  | | | Go to community leader (e.g. elder, clan chief, town chief) | | | | 1 | Go to religious leader | | | | | | | 9 | | |
|  | | | Go to justice of peace | | | | 2 | Go to a disabled people's organisation | | | | | | | 10 | | |
|  | | | Go to police | | | | 3 | Work with Human Rights organisation | | | | | | | 11 | | |
|  | | | Go to other family members | | | | 4 | Resort to the use of violence | | | | | | | 12 | | |
|  | | | Go to neighbour or friend | | | | 5 | Go to trial by ordeal (Sassy Wood) | | | | | | | 13 | | |
|  | | | Go to constituent leaders/ representatives | | | | 6 | Don't know | | | | | | | 88 | | |
|  | | | Go to district commissioner | | | | 7 | Did not answer | | | | | | | 99 | | |
|  | | | Go to court | | | | 8 | Other (please specify) | | | | | | | 14 | | |
| **H4_1** | How satisfied are you with your personal safety? ***[read out the options, circle one option]*** | | | **Not at all satisfied** | **A bit unsatisfied** | | | **neither satisfied or unsatisfied** | | | | **A bit satisfied** | **completely satisfied** | | | |  |
|  |  |  |  | 1 | 2 | | | 3 | | | | 4 | 5 | | | |  |
| **H4_2** | How satisfied are you with the safety of your household? ***[read out the options, circle one option]*** | | | **Not at all satisfied** | **A bit unsatisfied** | | | **neither satisfied or unsatisfied** | | | | **A bit satisfied** | **completely satisfied** | | | |  |
|  |  |  |  | 1 | 2 | | | 3 | | | | 4 | 5 | | | |  |
| **H4_3** | How satisfied are you with the safety in your community? ***[read out the options, circle one option]*** | | | 1 | 2 | | | 3 | | | | 4 | 5 | | | |  |

| **H4_4** | **Do you think you will feel more safe or less safe 1 year from now?** | | | |
| --- | --- | --- | --- | --- |
|  | More safe | 1 | Less safe | 3 |
|  | No change | 2 | Don’t know | 88 |
| **H4_5** | **Do you think you will feel more safe or less safe 5 years from now?** | | | |
|  | More safe | 1 | Less safe | 3 |
|  | No change | 2 | Don’t know | 88 |
| **H4_6** | **Do you think that you will be richer or poorer 1 year from now?** | | | |
|  | Richer | 1 | Poorer | 3 |
|  | No change | 2 | Don’t know | 88 |
| **H4_7** | **Do you think that you will be richer or poorer 5 years from now?** | | | |
|  | Richer | 1 | Poorer | 3 |
|  | No change | 2 | Don’t know | 88 |

**We have talked about a lot of different things today. I am now going to give you some cards with cover each of the themes. I would like you to put them in the order that you think they are the most important. I1**

| **Rank** | **Card #** |
| --- | --- |
| 1 |  |
| 2 |  |
| 3 |  |
| 4 |  |
| 5 |  |
| 6 |  |
| 7 |  |
| 8 |  |
| 9 |  |

**Thank you for taking the time to answer my questions.**
