# Supplementary material for: Inter- and intra-household perceived relative inequality among disabled and non-disabled people in Liberia
Source: PLoS One. 2019 Jul 17;14(7):e0217873. doi: 10.1371/journal.pone.0217873 (PMC6636711; doi:10.1371/journal.pone.0217873)
Supplement: S2 File — (DOCX) [file pone.0217873.s002.docx]

Table A. *Pearson correlations between age, sex, education and wealth quintile; by respondent type.*

| All respondents | Age (years) | Sex (m=0, f=1) | Education (1-9^a^) |
| --- | --- | --- | --- |
| Sex (male=0, female=1) | -0.0524* |  |  |
| Education (1-9 scale ^a^) | -0.2125**** | -0.3200**** |  |
| Wealth quintile (q1=poorest to q5=richest) | -0.1301**** | -0.1911**** | 0.3159**** |
| Household head in Disabled house | Age | Sex (m=0, f=1) | Education (1-9^a^) |
| Sex (male=0, female=1) | 0.0431 ns |  |  |
| Education (1-9 scale ^a^) | -0.1823* | -0.3014*** |  |
| Wealth quintile (q1=poorest to q5=richest) | -0.1712* | -0.3400**** | 0.3561**** |
| Person with Disabilities | Age | Sex (m=0, f=1) | Education (1-9^a^) |
| Sex (male=0, female=1) | 0.1984* |  |  |
| Education (1-9 scale^a^) | -0.2856*** | -0.2465** |  |
| Wealth quintile (q1=poorest to q5=richest) | -0.2055* | -0.0443 ns | 0.1090 ns |
| Other person in disabled house | Age | Sex (m=0, f=1) | Education (1-9^a^) |
| Sex (male=0, female=1) | 0.1596** |  |  |
| Education (1-9 scale ^a^) | -0.2748**** | -0.2710**** |  |
| Wealth quintile (q1=poorest to q5=richest) | -0.1299* | -0.0050 ns | 0.1080 ns |
| Head and person with Disabilities | Age | Sex (m=0, f=1) | Education (1-9^a^) |
| Sex (male=0, female=1) | -0.0110 ns |  |  |
| Education (1-9 scale ^a^) | -0.2462**** | -0.3564**** |  |
| Wealth quintile (q1=poorest to q5=richest) | -0.1179* | -0.2758**** | 0.4652**** |
| Household head in non-disabled house | Age | Sex (m=0, f=1) | Education (1-9^a^) |
| Sex (male=0, female=1) | -0.0657 ns |  |  |
| Education (1-9 scale ^a^) | -0.1940**** | -0.3891**** |  |
| Wealth quintile (q1=poorest to q5=richest) | -0.1293* | -0.2470**** | 0.4692**** |
| Matched respondent in non-disabled house | Age | Sex (m=0, f=1) | Education (1-9^a^) |
| Sex (male=0, female=1) | -0.0198 ns |  |  |
| Education (1-9 scale ^a^) | -0.2638**** | -0.2944**** |  |
| Wealth quintile (q1=poorest to q5=richest) | -0.0950* | -0.2090**** | 0.1843*** |

^a^ 1=No formal education; 2=Some primary; 3=Completed primary; 4=Some secondary; 5=Completed secondary; 6=Some college; 7=Completed college; 8=Some university; 9=University; ns=not significant **p*<0.05; ***p*<0.01; ****p*<0.001; *****p*<0.0001

Table B. *Associations between subjective satisfaction questions and age, sex, education and wealth quintile (regression coefficient, 95%CI, p-value).*

| Question ^a^ | Age (years) | Sex (m=0, f=1) | Education (1-9^b^) | Wealth quintile |
| --- | --- | --- | --- | --- |
| *A5: Life satisfaction:* Thinking about your own life and personal circumstances, how satisfied are you with your life as a whole? | -0.0066  (-0.0104, -0.0027)  *p*=0.001 | -0.3610  (-0.4873, -0.2348)  *p*<0.0001 | 0.1387  (0.1044, 0.1731)  *p*<0.0001 | 0.3139  (0.2710, 0.3568)  *p*<0.0001 |
| *B2_1: Living standards:* How satisfied are you with your own standard of living? | -0.0025  (-0.0061, 0.0011)  *p*=0.179 | -0.2099  (-0.3301, -0.0896)  *p*=0.001 | 0.1281  (0.0952, 0.1610)  *p*<0.0001 | 0.2723  (0.2313, 0.3133)  *p*<0.0001 |
| *C1: Health:* How satisfied are you with your health overall? | -0.0127  (-0.0167, -0.0088)  *p*<0.0001 | -0.0294  (-0.1614, 0.1025)  *p*=0.662 | 0.1163  (0.0803, 0.1523)  *p*<0.0001 | 0.2119  (0.1659, 0.2578)  *p*<0.0001 |
| *C4_1: Health access:* How satisfied are you with your access to health services? | -0.0094  (-0.1298, -0.0058)  *p*<0.0001 | -0.0344  (-0.1547, 0.859)  *p*=0.575 | 0.0633  (0.0303, 0.0962)  *p*<0.0001 | 0.2202  (0.1786, 0.2618)  *p*<0.0001 |
| *C4_13: Health* care: How satisfied are you with  the health care you receive? | -0.0083  (-0.0118, -0.0048)  *p*<0.0001 | -0.2467  (-0.3626, -0.1309)  *p*<0.0001 | 0.0998  (0.0681, 0.1315)  *p*<0.0001 | 0.2326  (0.1930, 0.2722)  *p*<0.0001 |
| *D3_1: Education:* How satisfied are you with the education/ school in your community? | -0.0012  (-0.0049, 0.0025)  *p*=0.540 | 0.1178  (-0.0027, 0.2382)  *p*=0.055 | -0.0640  (-0.0966, -0.0314)  *p*<0.0001 | 0.0385  (-0.0041, 0.0811)  *p*=0.076 |
| *E1_11: Work:* How satisfied are you with your work/employment? | -0.0011  (-0.0075, 0.0053)  *p*=0.732 | -0.1258  (-0.2985, 0.0470)  *p*=0.153 | 0.1060  (0.0622, 0.1499)  *p*<0.0001 | 0.1341  (0.0702, 0.1979)  *p*<0.0001 |
| *F1_1: Transport:* How satisfied are you with the access to transport in your community? | -0.0033  (-0.0069, 0.0004)  *p*=0.078 | -0.2355  (-0.3560, -0.1150)  *p*<0.0001 | 0.0718  (0.0387, 0.1049)  *p*<0.0001 | 0.1879  (0.1458, 0.2299)  *p*<0.0001 |
| *G2_1: Relationships with Friends:* How satisfied are you with your  relationships with friends? | 0.0001  (-0.0018, 0.0020)  0.931 | -0.0870  (-0.1493, -0.0246)  *p*=0.006 | 0.0358  (0.0190, 0.0525)  *p*<0.0001 | 0.0465  (0.0246, 0.0684)  *p*<0.0001 |
| *G2_2: Relationships with Household:* How satisfied are you with your  relationships with your household? | -0.0016  (-0.0032, -0.0000)  *p*=0.048 | -0.0446  (-0.0966, 0.0075)  *p*=0.093 | 0.0268  (0.0125, 0.0411)  *p*<0.0001 | 0.0546  (0.0361, 0.0731)  *p*<0.0001 |
| *G2_2: Relationship with Partner:* How satisfied are you with your  relationship with your husband/wife/partner? | 0.0028  (-0.0001, 0.0058)  *p*=0.058 | -0.0881  (-0.1748, -0.0014)  *p*=0.046 | 0.0142  (-0.0090, 0.0374)  *p*=0.230 | 0.0584  (0.0279, 0.0889)  *p*<0.0001 |
| *H4_1: Personal Safety:* How satisfied are you with your personal safety? | -0.0081  (-0.0111, -0.0052)  *p*<0.0001 | -0.0788  (-0.1784, 0.0207)  *p*=0.121 | 0.0262  (-0.0010, 0.0535)  *p*=0.059 | 0.0543  (0.0189, 0.0897)  *p*=0.003 |
| *H4_2: Household Safety:* How satisfied are you with the safety of your household? | -0.0000  (-0.0021, 0.0020)  *p*=0.973 | -0.0391  (-0.1078, 0.0297)  *p*=0.265 | -0.0068  (-0.0257, 0.0121)  *p*=0.480 | -0.0399  (-0.0640, -0.0158)  *p*=0.001 |
| *H4_3: Community Safety:* How satisfied are you with the safety of your community? | 0.0010  (-0.0015, 0.0035)  *p*=0.422 | 0.0046  (-0.0767, 0.0860)  *p*=0.912 | -0.0393  (-0.0616, -0.0171)  *p*=0.001 | -0.1006  (-0.1288, -0.0724)  *p*<0.0001 |

a All questions are asked as a 1-5 Likert scale: 1=Not at all satisfied; 2=a bit unsatisfied; 3=not satisfied or unsatisfied; 4=a bit satisfied; 5=completely satisfied

b 1=No formal education; 2=Some primary; 3=Completed primary; 4=Some secondary; 5=Completed secondary; 6=Some college; 7=Completed college; 8=Some university; 9=University

Regression coefficients are change in 5-point Likert scale per unit increase in age, sex, education or wealth quintile.

Table C. *Associations between objective questions and age, sex, education and wealth quintile (regression coefficient, 95%CI, p-value).*

| Question ^a^ | Age (years) | Sex (m=0, f=1) | Education (1-9^b^) | Wealth quintile |
| --- | --- | --- | --- | --- |
| *C4_2: Getting needed Healthcare:* How often can you get the healthcare you need?^a^ | 0.0039  (0.0018, 0.0060)  *p*< 0.0001 | -0.0320  (-0.1008, 0.0368)  *p*=0.362 | -0.0116  (-0.0306, 0.0073)  *p*=0.230 | -0.0133  (-0.0380, 0.0113)  *p*=0.289 |
| *D1: Education:* What is the highest level of education you have completed? ^b^ | -0.0235  (-0.0282, -0.0187)  *p*< 0.0001 | -1.171  (-1.322, -1.0185)  *p*<0.0001 | N/A | 0.3961  (0.3426, 0.4496)  *p*<0.0001 |
| *E1_4: Income:* How much money do you make per month? (Liberian $) | 142  (21, 263)  *p*= 0.022 | -7581  (-11051, -4111)  *p* < 0.0001 | 2843  (1981, 3706)  *p*<0.0001 | 3195  (1853, 4537)  *p*<0.0001 |
| *F1_3 Transport Access:* How often do you have access to the transport you need? ^a^ | -0. 0032  (-0.0058, -0.0006)  *p*= 0.015 | -0.1543  (-0.2391, -0.0695)  *p* < 0.0001 | 0.0358  (0.0126, 0.0591)  *p*=0.003 | 0.0982  (0.6796, 0.1285)  *p*<0.0001 |
| *G4_1 Vote:* Do you vote? ^c^ | 0. 0044  (0.0036, 0.0051)  *p*<0.0001 | -0.2467  (-0.3626, -0.1309)  *p*<0.0001 | 0.0026  (-0.0044, 0.0096)  *p*=0.463 | -0.0054  (-0.0145, 0.0037)  *p*=0.246 |
| *H1_3 & H1_5: Crime:* Have you personally experienced any form of crime or violence in the last year? Has anyone in your household witnessed any crime or violence in the last year? ^d^ | -0.0015  (-0.0028, 0.0003)  *p*=0.017 | -0.0272  (-0.0529, -0.0014)  *p*=0.038 | 0.0109  (-0.0005, 0.0222)  *p*=0.060 | 0.0202  (0.0052, 0.0353)  *p*=0.008 |

^a^ Coded on a 4 point scale: 1=Never; 2=Occasionally/Sometimes; 3=Most of the time; 4= All of the time

^b^ Coded on a 9-point scale: 1=No formal education; 2=Some primary; 3=Completed primary; 4=Some secondary; 5=Completed secondary; 6=Some college; 7=Completed college; 8=Some university; 9=University. Note this model, with education as the outcome, unlike the other models obviously did not include education as an explanatory variable.

^c^ 1=Yes (sometimes or always); 0=No; the two respondents who refused the question were coded as missing

^d^ 1=Yes (once, or more than once for personally experienced crime, or household member witnessed a crime); 0=No (not experienced crime in the past year); 'don't know' (88; 7%) and 'refused answer' (99; 3%) to question H1_3 recoded as missing

Regression coefficients are change in 5-point Likert scale per unit increase in age, sex, education or wealth quintile.

Table D. *Intracorrelations in subjective satisfaction indicators and their intercorrelations with objective and community relations indicators.*

| Variable | 1 | 2 | 3 | 4 | 5 | 6 | 7 | 8 | 9 | 10 | 11 | 12 | 13 | 14 |
| --- | --- | --- | --- | --- | --- | --- | --- | --- | --- | --- | --- | --- | --- | --- |
| Subjective |  |  |  |  |  |  |  |  |  |  |  |  |  |  |
| 1.Life Satisfaction | - |  |  |  |  |  |  |  |  |  |  |  |  |  |
| 2.Living Standards | .73*** | - |  |  |  |  |  |  |  |  |  |  |  |  |
| 3.Health | .45*** | .43*** | - |  |  |  |  |  |  |  |  |  |  |  |
| 4.Health access | .45*** | .45*** | .51*** | - |  |  |  |  |  |  |  |  |  |  |
| 5. Healthcare | .43*** | .43*** | .46*** | .65*** | - |  |  |  |  |  |  |  |  |  |
| 6. Education | .10*** | .11*** | 12*** | .23*** | .22*** | - |  |  |  |  |  |  |  |  |
| 7. Work | .28*** | .36*** | 17*** | .26*** | .28*** | .07 | - |  |  |  |  |  |  |  |
| 8. Transport | .37*** | .39*** | .29*** | .31*** | .36*** | .11*** | .25*** | - |  |  |  |  |  |  |
| 9. Relationships with Friends | .15*** | .14*** | .13*** | .09*** | .13*** | -.10*** | .09* | .08*** | - |  |  |  |  |  |
| 10. Relationships with Household | .20*** | .15*** | 19*** | .18*** | .18*** | -.01 | .08* | -.01 | .37*** | - |  |  |  |  |
| 11. Relationships with Partner | .16*** | .08** | .06* | .04 | .06 | .03 | .12** | .02 | .11*** | .34*** | - |  |  |  |
| 12. Personal Safety | .16*** | .14*** | .25*** | .21*** | .20*** | .08*** | .14*** | .03 | .13*** | .29*** | .20*** | - |  |  |
| 13. Household Safety | .02 | .05* | .09*** | .04 | .01 | .02 | .06 | -.04 | .06* | .22*** | .16*** | .64*** | - |  |
| 14. Community Safety | -.06** | -0.08** | .06* | -.02 | -.04 | -.01 | .00 | .15*** | .05* | .11*** | .08** | .51*** | .64*** | - |

| Objective |  |  |  |  |  |  |  |  |  |  |  |  |  |  |
| --- | --- | --- | --- | --- | --- | --- | --- | --- | --- | --- | --- | --- | --- | --- |
| 15. Getting Healthcare | .03 | .04 | .03 | .18*** | .14*** | .09*** | .01 | .14*** | .01 | -.04 | -.03 | -.03 | .03 | .01 |
| 16. Education | .18*** | .17*** | .14*** | .08*** | .14*** | -.09*** | .17*** | .10*** | .10*** | .08*** | .04 | .04 | -.02 | -.08*** |
| 17. Income | .19*** | .23*** | .04 | .06 | .10* | -.07 | .21*** | .16*** | .07 | .05 | .03 | .06 | .01 | .02 |
| 18. Transport | .26*** | .28*** | .19*** | .21*** | .21*** | .01 | .21*** | .50*** | .03 | .04 | .06 | .13 | .02 | -.11 |
| 19. Voting | .03 | .00 | .05* | -.05* | -.01 | -.08 | -.05 | .03 | .01 | -.00 | .03 | -.01 | .04 | .03 |
| 20. Crime | .04 | .04 | .03 | .05* | .09*** | -.01 | .01 | .12*** | -.01 | .02 | -.10** | -.22*** | -.25*** | -.26*** |
| Community Relations |  |  |  |  |  |  |  |  |  |  |  |  |  |  |
| 21. Community Inclusion | .05* | .06* | .11*** | .01 | .02 | -.11*** | .11** | -.04* | .12*** | .09*** | .01 | .10*** | .10*** | .16*** |
| 22. Community Participation | .02 | .01 | .10*** | .01 | .01 | -.03 | .02 | -.01 | .10*** | .08*** | .04 | 12*** | .14*** | .14*** |
| 23. Friends | -.04 | -.04 | .03 | .-10*** | -.05* | -.04 | .09** | -.03 | .08*** | .01 | -.01 | .04 | .06** | .06** |
| 24. Getting Help from Community | .01 | .04 | .09*** | .08*** | .07** | .16*** | .18*** | .03 | .01 | .03 | -.00 | .14*** | .17*** | .21*** |
| 25. Giving Help to Community | .12*** | .12*** | .19*** | .15*** | .16*** | .09*** | .16*** | .07** | .05* | .07** | .04 | .16*** | .13*** | .15*** |
| 26. Trust | .08** | .07** | .07** | .07** | .09*** | .00 | .05 | .08*** | .15*** | .11*** | .11*** | .14*** | .13*** | .15*** |
| 27. Inclusion in Decision Making | .07** | .07** | .11*** | .02 | .02 | -.11*** | .08* | -.01 | .12*** | .07** | .01 | .07** | .08*** | .16*** |

*Note*. *N* range = 395 to 1093 for correlations with Transport, Relationships with Partner, and Income; all other correlations *N* range = 1679 to 2002 * *p* < .05, ** *p* < .01, *** *p* < .001.

Table E. *Intracorrelations in objective and community relations indicators and their intercorrelations.*

| Variable | 1 | 2 | 3 | 4 | 5 | 6 | 7 | 8 | 9 | 10 | 11 | 12 | 13 |
| --- | --- | --- | --- | --- | --- | --- | --- | --- | --- | --- | --- | --- | --- |
| Objective |  |  |  |  |  |  |  |  |  |  |  |  |  |
| 1.Getting Healthcare | - |  |  |  |  |  |  |  |  |  |  |  |  |
| 2.Education | -.03 | - |  |  |  |  |  |  |  |  |  |  |  |
| 3.Income | .03 | .25*** | - |  |  |  |  |  |  |  |  |  |  |
| 4.Transport | .20*** | .07** | .14*** | - |  |  |  |  |  |  |  |  |  |
| 5.Voting | .03 | .02 | .09* | .07** | - |  |  |  |  |  |  |  |  |
| 6. Crime | .00 | .04 | .02 | .10*** | -.03 | - |  |  |  |  |  |  |  |
| Community Relations |  |  |  |  |  |  |  |  |  |  |  |  |  |
| 7. Community Inclusion | -.05* | .15*** | .08* | -.06** | .16*** | -.04 | - |  |  |  |  |  |  |
| 8. Community Participation | .03 | .05* | -.01 | -.01 | .15*** | -.02 | .45*** | - |  |  |  |  |  |
| 9. Friends | -.02 | .08*** | .06 | -.02 | .12*** | .01 | .26*** | .21*** | - |  |  |  |  |
| 10. Getting Help from Community | .12*** | -.00 | -.01 | .08*** | .06* | -.06* | .16*** | .24*** | .14*** | - |  |  |  |
| 11. Giving Help to Community | .11*** | .11*** | .11** | .13*** | .13*** | -.02 | .25*** | .33*** | .14*** | .74*** | - |  |  |
| 12. Trust | .03 | .00 | .04 | .09*** | .06* | -.05* | .12*** | .09*** | .03 | .17*** | .19*** | - |  |
| 13. Inclusion in Decision Making | -.04 | .11*** | .09* | -.04 | .25*** | -.03 | .77*** | .46*** | .25*** | .22*** | .29*** | .14*** | - |

*Note.* *N* range = 630 to 649 for correlations with Income; all other intracorrelations *N* range = 1761 to 2007. * *p* < .05, ** *p* < .01, *** *p* < .001

Table F. *Associations between all other Likert questions and respondent type, adjusted for age, sex, education and wealth quintile, and clustering by household, village and county (regression coefficient, 95%CI, p-value).*

| Comparison | A (ref group: 6. age and sex matched in non-disabled household) | | B (ref group: 3. Other non-disabled in disabled household) | | C (ref group: 1. head of household in disabled household) | D (ref group: 6. age and sex matched in non-disabled household) |
| --- | --- | --- | --- | --- | --- | --- |
| Question | 2. Disabled | 4. Head of household and Disabled | 2. Disabled | 4. Head of household and Disabled | 2. Disabled | 5. Head of household (non-disabled house) |
| *B1_4: Safe Drinking Water:* How safe is it for women, children, persons with disabilities and elders to reach to the nearest supply of drinking water?^a^ | -0.007  (-0.217, 0.203)  *p*=0.949 | -0.195  (-0.382, -0.008)  *p*=0.041 | -0.075  (-0.295, 0.146)  *p*=0.506 | -0.263  (-0.473, -0.052)  *p*=0.014 | 0.037  (-0.238, 0.311)  *p*=0.793 | -0.524  (-0.672, -0.376)  *p*<0.0001 |
| *B2_2: Living Well due to Income:* How well do you live based on your current household income? ^b^ | -0.572  (-0.742, -0.401)  *p*<0.0001 | -0.493  (-0.626, -0.360)  *p*<0.0001 | -0.577  (-0.755, -0.398)  *p*<0.0001 | -0.498  (-0.650, -0.347)  *p*<0.0001 | -0.497  (-0.694, -0.230)  *p*<0.0001 | 0.148  (0.038, 0.259)  *p*=0.009 |
| *B2_3: Relative Living Standard*: How would you compare your standard of living to other households in your community?^c^ | -0.414  (-0.564, -0.264)  *p*<0.0001 | -0.316  (-0.433, -0.198)  *p*<0.0001 | -0.476  (-0.633, -0.318)  *p*<0.0001 | -0.377  (-0.512, -0.243)  *p*<0.0001 | -0.709  (-0.886, -0.533)  *p*<0.0001 | 0.298  (0.199, 0.396)  *p*<0.0001 |
| D2_1: *Reading Ability:* Can you read and write?^d^ | -0.053  (-0.183, 0.077)  *p*=0.424 | -0.212  (-0.318, -0.106)  p<0.0001 | -0.108  (-0.245, 0.029)  *p*=0.122 | -0.267  (-0.254, -0.035)  *p*<0.0001 | -0.049  (-0.107, 0.206)  *p*=0.537 | -0.090  (-0.318, -0.106)  *p*=0.051 |
| D2_2: *Math Ability:* Can you do maths/sums?^d^ | -0.045  (-0.176, 0.087)  *p*=0.504 | -0.271  (-0.379, -0.163)  *p*<0.0001 | -0.068  (-0.206, 0.070)  *p*=0.337 | -0.294  (-0.416, -0.171)  *p*<0.0001 | -0.092  (-0.066, 0.250)  *p*=0.253 | -0.157  (-0.251, -0.062)  *p*=0.001 |
| *D3_2: General Importance of Education:* How important do you think it is for a child to go to school?^e^ | -0.023  (-0.103, 0.057)  *p*=0.571 | -0.058  (-0.120. 0.004)  *p*=0.065 | 0.070  (-0.014, 0.154)  *p*=0.102 | 0.035  (-0.034, 0.104)  *p*=0.317 | 0.030  (-0.059, 0.119)  *p*=0.513 | -0.016  (-0.065, 0.034)  *p*=0.541 |
| *D3_3:* *Importance of Education for Disabled People:* How important is education for persons with disabilities?^e^ | 0.082  (-0.007, 0.171)  *p*=0.071 | 0.035  (-0.035, 0.104)  *p*=0.327 | 0.046  (-0.048, 0.139)  *p*=0.339 | -0.002  (-0.079, 0.075)  *p*=0.958 | -0.008  (-0.109, 0.093)  *p*=0.883 | -0.066  (-0.123, -0.009)  *p*=0.022 |

**^a^** Coded on a 5 point scale**:** 1**=**Very unsafe; 2= A bit unsafe; 3=Neither safe nor unsafe; 4=A bit safe; 5=Very safe

^b^ Coded on a 5 point scale**:** 1**=**Living with a lot of difficulty; 2=Living with a bit of difficulty; 3=Managing; 4=Living a bit well ; 5=Living very well

^c^ Coded on a 5 point scale**:** 1**=**Poorer; 2=Fairly poor; 3=In the middle; 4= Fairly rich ; 5=Richer

^d^ Coded on a 4 point scale: 1 = Cannot do at all; 2=With a lot of difficulty; 3=With some difficulty; 4=Well

^e^ Coded on a 5 point scale**:** 1**=**Very unimportant; 2=A bit unimportant; 3=neither important or unimportant; 4=A bit important; 5=Very important

*Summary of findings in Appendix 1:* Looking at the correlations between potential confounding variables (Supplementary Table 1) female respondents are slightly younger in the overall sample, but among persons with disabilities (group 2), or non-disabled members of disabled households (group 3) specifically females are more likely to be older overall. Respondents with higher educational achievement are more likely to be younger, and male. This was found to be the case in all comparison groups of respondents. Wealthier respondents are more likely to be younger, male, and more educated. This was found to be the case in all groups of respondents except persons with disabilities (group 2), and other people in disabled households (group 3) – in these groups there was still a negative correlation between wealth and age, due to wealth being calculated at the household level (i.e. the same for all members in the disabled household). Supplementary Tables 2a & 2b detail, respectively, the associations between each of the 14 subjective satisfaction indicators, and the six objective indicators; and age, sex, education and wealth quintile. In general, across the subjective satisfaction indicators, younger, male, more educated, and wealthier respondents reported higher satisfaction, with wealth correlated significantly with all indicators. Interestingly, wealth was associated with greater satisfaction with personal safety, but lower satisfaction with household and community safety, indicating perhaps respondents with more possessions to loose perceived themselves most at risk. Across the objective indicators, older respondents reported getting needed healthcare more frequently and greater voter participation, while males reported a higher income, greater transport access and greater voter participation. Both higher educational achievement and wealth quintile were associated with greater income and transport access, and wealth was additionally associated with a greater likelihood of experiencing crime.

We also checked intra- and intercorrelations between the subjective and objective indicators (Supplementary 3a and 3b). As might be expected, the majority of subjective satisfaction indicators were significantly and positively intracorrelated and subjective satisfaction indicators generally possessed significant and positive intercorrelations with objective indicators in the same domain (e.g., health and so forth). An exception was satisfaction with education in the community, which correlated negatively with educational achievement (i.e. better educated respondents were less satisfied with education in their community). Among the objective indicators, greater access to transport was positively associated with all other indicators, including likelihood of experiencing crime.

We also checked associations between respondent type and all other Likert type questions in our study that were not of central interest to our research questions (Supplementary Table 4). Compared to non-disabled Liberians in the same and other households (groups 1, 3 and 6) disabled Liberians, including household heads (groups 2 and 4) reported living less well due to income and a poorer relative living standard (all *p* < 0.0001). Conversely, non-disabled household heads reported living better due to income (*p* = .009) and a better relative living standard (at *p* < 0.0001) compared to other non-disabled members of their households. These findings are in line with those reported in the main manuscript regarding subjective life satisfaction and living standards. Interestingly, disabled household heads also reported poorer reading and math ability compared to non-disabled Liberians in the same and other households (all *p* <0.0001) whereas other disabled Liberians did not (*p* >.423).
