# Supplementary material for: Inter- and intra-household perceived relative inequality among disabled and non-disabled people in Liberia
Source: PLoS One. 2019 Jul 17;14(7):e0217873. doi: 10.1371/journal.pone.0217873 (PMC6636711; doi:10.1371/journal.pone.0217873)
Supplement: S3 File — (DOCX) [file pone.0217873.s003.docx]

As we identified disabled people in our study via DPO lists, we sought to cross-check the disability status of these respondents via analysis using the Washington Group Short Set for adults. The set is not a diagnostic tool for disability, but assesses functional difficulty across six domains (seeing, hearing, walking, concentration, self-care, and understanding: see Appendix 2, section C2 of the questionnaire,). Thus, we expected disability as identified via the conventional Washington Group cut-off (i.e. a response of “with a lot of difficulty” or “cannot do at all” in any one domain), to correspond closely, but not perfectly, to the DPO lists. Our analyses bore this prediction out. Specifically, 80% of the identified disabled respondents (*N* = 395) met the Washington Group criteria for disability (75% of disabled respondents, *N* = 123; and 82% of disabled household heads, *N* = 272). We also identified 5% (*N* = 70) of non-DPO list (i.e. non-disabled) respondents as meeting the Washington Group criteria for disability (12% of household heads in disabled households, *N* = 18; 8% of other respondents in disabled households, *N* = 29; 2% of household heads in control households, *N* = 9; and 3% of age- and sex-matched respondents in control households, *N* = 13)^^[[1]](#footnote-1)^^

We reran the main comparisons in our study dropping the 70 cases from the non-DPO list that were picked up as disabled by the Washington Group questions. Results are shown in Supplementary Tables 5, 6 and 7 and our findings did not change in the vast majority of cases. Five results which were borderline non-significant (i.e. *p* = .001 or *p* = .002) became significant, whereas one became borderline non-significant (*p* = 0.001). Four of these six differences were comparisons for the subjective questions pertaining to relationships or safety.

These key differences are highlighted below:

1. Disabled respondents (group 2) less satisfied with access to health services (*p* < 0.001) compared to non-disabled heads of their households (group 1). Previously *p* = 0.001.
2. Disabled household heads (group 4) less satisfied with relationships in their household (*p* < 0.001) compared to other non-disabled members of their households (group 3). Previously *p* = 0.001.
3. No difference in satisfaction with relationship with partner (*p* = 0.001) between disabled household heads (group 4) and non-disabled respondents in other households (group 6). Previously *p* = 0.001.
4. Disabled respondents (group 2) less satisfied with personal safety (*p* = 0.001) compared to compared to other non-disabled members of their households (group 3). Previously *p* = 0.001.
5. Disabled household heads (group 4) less satisfied with their household safety (*p* < 0.001) compared to other non-disabled members of their households (group 3). Previously *p* = 0.001.
6. Disabled respondents (group 2) report lower educational attainment (*p* = 0.001) compared to non-disabled heads of their households (group 1). Previously *p* = 0.002.

Table A. *Associations between subjective satisfaction questions and respondent type (Washington Group identified), adjusted for age, sex, education and wealth quintile, and clustering by household, village and county (regression coefficient, 95%CI, p-value).*

| Comparison | A (ref group: 6. age and sex matched in non-disabled household) | | B (ref group: 3. Other non-disabled in disabled household) | | C (ref group: 1. head of household in disabled household) | D (ref group: 6. age and sex matched in non-disabled household) |
| --- | --- | --- | --- | --- | --- | --- |
| Question ^a^ | 2. Disabled | 4. Head of household and Disabled | 2. Disabled | 4. Head of household and Disabled | 2. Disabled | 5. Head of household (non-disabled house) |
| *A5: Life satisfaction:* Thinking about your own life and personal circumstances, how satisfied are you with your life as a whole? | -0.987  (-1.214, -0.761)  *p*<0.0001 | -0.723  (-0.897, -0.549)  *p*<0.0001 | -0.918  (-1.159, -0.677)  *p*<0.0001 | -0.654  (-0.857, -0.450)  *p*<0.0001 | -1.043  (-1.304, -0.782)  *p*<0.0001 | 0.279  (0.136, 0.422)  *p*<0.001 |
| *B2_1: Living standards:* How satisfied are you with your own standard of living? | -0.953  (-1.179, -0.727)  *p*<0.0001 | -0.649  (-0.825, -0.472)  *p*<0.0001 | -0.978  (-1.122, -0.739)  *p*<0.0001 | -0.674  (-0.875, -0.472)  *p*<0.0001 | -0.761  (-1.023, -0.499)  *p*<0.0001 | 0.302  (0.158, 0.446)  *p*<0.0001 |
| *C1: Health:* How satisfied are you with your health overall? | -1.343  (-1.585, -1.100)  *p*<0.0001 | -1.173  (-1.363, -0.983)  *p*<0.0001 | -1.694  (-1.953, -1.436)  *p*<0.0001 | -1.525  (-1.748, -1.300)  *p*<0.0001 | -1.527  (-1.824, -1.230)  *p*<0.0001 | -0.131  (-0.293, 0.031)  *p*=0.114 |
| *C4_1: Health access:* How satisfied are you with your access to health services? | -0.204  (-0.436, 0.028)  *p*=0.084 | -0.510  (-0.691, -0.329)  *p*<0.0001 | -0.783  (-1.026, -0.540)  *p*<0.0001 | -1.089  (-1.294, -0.883)  *p*<0.0001 | -0.565  (-829, -0.300)  *p*<0.001 | 0.150  (0.004, 0.296)  *p*=0.045 |
| *C4_13: Health care*: How satisfied are you with  the health care you receive?^b^ | -0.110  (-0.326, 0.105)  *p*=0.316 | -0.226  (-0.394, -0.058)  *p*=0.009 | -0.587  (-0.812, -0.362)  *p* < 0.0001 | -0.702  (-0.891, -0.513)  *p*<0.0001 | -0.379  (-0.622, -0.136)  *p*=0.002 | 0.140  (0.005, 0.275)  *p*=0.043 |
| *D3_1: Education:* How satisfied are you with the education/ school in your community? | 0.806  (0.555, 1.057)  *p*<0.0001 | 0.429  (0.235, 0.622)  *p*<0.0001 | 0.081  (-0.344, 0.182)  *p*=0.545 | -0.459  (-0.684, -0.234)  *p*<0.0001 | 0.128  (-0.169, 0.424)  *p*=0.398 | 0.351  (0.186, 0.516)  *p*<0.0001 |
| *E1_11: Work:* How satisfied are you with your work/employment? | 0.050  (-0.420, 0.519)  *p*=0.835 | -0.403  (-0.709, -0.097)  *p*=0.010 | -0.201  (-0.675, 0.272)  *p*=0.405 | -0.654  (-0.995, -0.313)  *p*<0.001 | 0.079  (-0.436, 0.594)  *p*=0.763 | 0.208  (-0.017, 0.434)  *p*=0.071 |
| *F1_1: Transport:* How satisfied are you with the access to transport in your community? | -0.496  (-0.739, -0.254)  *p*<0.0001 | -0.067  (-0.257, 0.123)  *p*=0.491 | -0.529  (-0.788, -0.269)  *p*<0.0001 | -0.099  (-0.324, 0.126)  *p*=0.389 | -0.939  (-1.123, -0.645)  *p*<0.0001 | 0.469  (0.306, 0.631)  *p*<0.0001 |
| *G2_1: Relationships with Friends:* How satisfied are you with your  relationships with friends? | -0.252  (-0.380, -0.124)  *p*<0.001 | -0.170  (-0.269, -0.071)  *p*=0.001 | -0.130  (-0.267, 0.008)  *p* = 0.154 | -0.048  (-0.166, 0.070)  *p*=0.427 | -0.126  (-0.140, 0.149)  *p*=0.124 | -0.173  (-0.260, -0.087)  *p*<0.0001 |
| *G2_2: Relationships with Household:* How satisfied are you with your  relationships with your household? | -0.102  (-0.211, 0.007)  *p*=0.067 | -0.227  (-0.313, -0.141)  *p*<0.0001 | -0.061  (-0.179, 0.056)  *p*=0.309 | -0.186  (-0.289, -0.084)  *p*<0.001 | 0.029  (-0.105, 0.164)  *p*=0.667 | -0.166  (-0.240, -0.092)  *p*<0.0001 |
| *G2_3: Relationship with Partner:* How satisfied are you with your  relationship with your husband/wife/partner? | -0.137  (-0.366, 0.092)  *p*=0.242 | -0.256  (-0.401, -0.111)  *p*=0.001 | 0.170  (-0.071, 0.412)  *p*=0.167 | 0.051  (-0.122, 0.224)  *p*=0.562 | -0.014  (-0.263, 0.236)  *p*=0.914 | -0.172  (-0.284, -0.059)  *p*=0.003 |
| *H4_1: Personal Safety:* How satisfied are you with your personal safety? | -0.555  (-0.750, -0.360)  *p*<0.0001 | -0.647  (-0.799, -0.494)  *p*<0.0001 | -0.402  (-0.611, -0.193)  *p*<0.001 | -0.494  (-0.675, -0.312)  *p*<0.0001 | -0.276  (-0.516, -0.036)  *p*=0.024 | -0.879  (-1.011, -0.748)  *p*<0.0001 |
| *H4_2: Household Safety:* How satisfied are you with the safety of your household? | -0.276  (-0.418, -0.133)  *p*<0.001 | -0.349  (-0.460, -0.238)  *p*<0.0001 | -0.182  (-0.333, -0.030)  *p*=0.019 | -0.255  (-0.385, -0.125)  *p*<0.001 | -0.119  (-0.291, 0.052)  *p*=0.171 | -0.250  (-0.344, -0.156)  *p*<0.0001 |
| *H4_3: Community Safety:* How satisfied are you with the safety of your community? | -0.296  (-0.458, -0.135)  *p*<0.001 | -0.452  (-0.578, -0.326)  *p*<0.0001 | -0.262  (-0.434, -0.091)  *p*=0.003 | -0.418  (-0.564, -0.271)  *p*<0.0001 | -0.070  (-0.264, 0.123)  *p*=0.475 | -0.356  (-0.462, -0.250)  *p*<0.0001 |

^a^ All questions are asked as a 1-5 Likert scale: 1=Not at all satisfied; 2=a bit unsatisfied; 3=not satisfied or unsatisfied; 4=a bit satisfied; 5=completely satisfied

Regression coefficients are difference in 5-point Likert scale relative to comparison group 6: respondents matched on age and sex with disabled respondents. Given multiple comparisons *p*-values above 0.001 are not considered significant

Table B. *Associations between objective questions and respondent type (Washington Group identified), adjusted for age, sex, education and wealth quintile and clustering by household, village and county (regression coefficient, 95%CI, p-value).*

| Comparison | A (ref group: 6. age and sex matched in non-disabled household) | | B (ref group: 3. Other non-disabled in disabled household) | | C (ref group: 1. head of household in disabled household) | D (ref group: 6. age and sex matched in non-disabled household) |
| --- | --- | --- | --- | --- | --- | --- |
| Question ^a^ | 2. Disabled | 4. Head of household and Disabled | 2. Disabled | 4. Head of household and Disabled | 2. Disabled | 5. Head of household (non-disabled house) |
| *C4_2: Getting needed Healthcare:* How often can you get the healthcare you need?^a^ | -0.228  (-0.369, -0.087)  *p*=0.002 | 0.369  (0.259, 0.479)  *p*<0.0001 | -0.109  (-0.259, 0.042)  *p*=0.157 | 0.488  (0.358, 0.618)  *p*<0.0001 | -0.873  (-1.044, -0.702)  *p*<0.0001 | 0.131  (0.037, 0.225)  *p*=0.006 |
| *D1: Education:* What is the highest level of education you have completed? ^b^ | -0.332  (-0.628, -0.036)  *p*=0.028 | 0.006  (-0.227, 0.238)  *p*=0.963 | -0.332  (-0.645, -0.018)  *p*=0.038 | 0.006  (-0.257, 0.269)  *p*=0.966 | -0.552  (-0.891, -0.213)  *p*=0.001 | 0.082  (-0.105, 0.269)  *p*=0.389 |
| *E1_4: Income:* How much money do you make per month? (Liberian $) | -1218  (-9565, 7128)  *p*=0.775 | -1979  (-7820, 3860)  *p*=0.506 | 681  (-7494, 8856)  *p*=0.870 | -80  (-6485, 6325)  *p*=0.980 | -9614  (-19774, 545)  *p*=0.064 | 5498  (1284, 9713)  *p*=0.011 |
| *F1_3 Transport Access:* How often do you have access to the transport you need? ^a^ | -0.700  (-0.870, -0.529)  *p*<0.0001 | -0.035  (-0.169, 0.098)  *p*=0.602 | -0.422  (-0.606, -0.239)  *p*<0.0001 | 0.242  (0.083, 0.401)  *p*=0.003 | -1.052  (-1.261, -0.843)  *p*<0.0001 | 0.034  (-0.081, 0.149)  *p*=0.558 |
| *G4_1 Vote:* Do you vote? ^c^ | -0.251  (-0.300, -0.200)  *p*<0.0001 | -0.0004  (-0.040, 0.039)  *p*=0.982 | -0.162  (-0.217, -0.108)  *p*<0.0001 | 0.087  (0.041, 0.134)  *p*<0.0001 | -0.285  (-0.345, -0.224)  *p*<0.0001 | 0.007  (-0.026, 0.041)  *p*=0.662 |
| *H1_3 & H1_5: Crime:* Have you personally experienced any form of crime or violence in the last year? Has anyone in your household witnessed any crime or violence in the last year? ^d^ | 0.129  (0.042, 0.216)  *p*=0.003 | 0.188  (0.122, 0.254)  *p*<0.0001 | 0.038  (-0.056, 0.132)  *p*=0.428 | 0.097  (0.018, 0.175)  *p*=0.016 | -0.028  (-0.130, 0.074)  *p*=0.590 | 0.106  (0.046, 0.166)  *p*<0.001 |

^a^ Coded on a 4 point scale: 1=Never; 2=Occasionally/Sometimes; 3=Most of the time; 4= All of the time

^b^ Coded on a 9-point scale: 1=No formal education; 2=Some primary; 3=Completed primary; 4=Some secondary; 5=Completed secondary; 6=Some college; 7=Completed college; 8=Some university; 9=University. Note this model, with education as the outcome, unlike the other models obviously did not include education as an explanatory variable.

^c^ 1=Yes (sometimes or always); 0=No; the two respondents who refused the question were coded as missing

^d^ 1=Yes (once, or more than once for personally experienced crime, or household member witnessed a crime); 0=No (not experienced crime in the past year); 'don't know' (88; 7%) and 'refused answer' (99; 3%) to question H1_3 recoded as missing

Given multiple comparisons testing *p*-values above 0.001 are not considered significant

Table C. *Associations between community relations indicators and respondent type (Washington Group identified), adjusted for age, sex, education and wealth quintile, and clustering by household, village and county (regression coefficient, 95% CI, p-value).*

| Comparison | A (ref group: 6. age and sex matched in non-disabled household) | | B (ref group: 3. Other non-disabled in disabled household) | | C (ref group: 1. head of household in disabled household) | D (ref group: 6. age and sex matched in non-disabled household) |
| --- | --- | --- | --- | --- | --- | --- |
| Question | 2. Disabled | 4. Head of household and Disabled | 2. Disabled | 4. Head of household and Disabled | 2. Disabled | 5. Head of household (non-disabled house) |
| *G1_1: Community Inclusion:* How included do you feel in your community?^a^ | -0.787  (-1.028, -0.545)  *p*<0.0001 | -0.664  (-0.854, -0.475)  *p*<0.0001 | -0.628  (-0.887, -0.369)  *p*<0.0001 | -0.506  (-0.730, -0.282)  *p*<0.0001 | -0.589  (-0.884, -0.295)  *p*<0.0001 | -0.205  (-0.367, -0.043)  *p*=0.013 |
| *G1_3: Community Participation:* Do you participate in any community activities? ^b^ | -0.412  (-0.494, -0.330)  *p*<0.0001 | -0.165  (-0.230, -0.101)  *p*<0.0001 | -0.355  (-0.443, -0.266)  *p*<0.0001 | -0.108  (-0.185, -0.031)  *p*=0.006 | -0.392  (-0.494, -0.291)  *p*<0.0001 | -0.032  -0.087, 0.024  *p*=0.263 |
| *G1_6: Friends*: Do you have friends?^c^ | -0.120  (-0.219, -0.031)  *p*=0.008 | 0.071  (0.002, 0.141)  *p*=0.045 | -0.223  (-0.318, -0.128)  *p*<0.0001 | -0.032  (-0.114, 0.050)  *p*=0.447 | -0.280  (-0.387, -0.173)  *p*<0.0001 | -0.019  (-0.078, 0.040)  *p*=0.535 |
| *G3_1 & G3_3: Getting Help from Community:* Do your neighbours help you when you ask for assistance? Does your community help when you ask for assistance?^d^ | -0.144  (-0.280, -0.008)  *p*=0.038 | -0.035  (-0.141, 0.071)  *p*=0.515 | -0.418  (-0.562, -0.274)  *p*<0.0001 | -0.309  (-0.432, -0.186)  *p*<0.0001 | -0.279  (-0.440, -0.119)  *p*=0.001 | 0.046  (-0.042, 0.134)  *p*=0.305 |
| G3_2 & G3_4*: Giving Help to Community:* Do you help your neighbours when they ask for assistance? Do you help your community in community initiatives?^d^ | -0.358  (-0.489, -0.227)  *p*<0.0001 | -0.055  (-0.156, 0.047)  *p*=0.292 | -0.609  (-0.749, -0.469)  *p*<0.0001 | -0.305  (-0.425, -0.185)  *p*<0.0001 | -0.633  (-0.791, -0.474)  *p*<0.0001 | 0.071  (-0.016, 0.157)  *p*=0.109 |
| G3_5: *Trust:* How much do you trust your neighbours?^e^ | -0.451  (-0.637, -0.264)  *p*<0.0001 | -0.303  (-0.448, -0.158)  *p*<0.0001 | -0.270  (-0.471, -0.068)  *p*=0.009 | -0.122  (-0.293, 0.049)  *p*=0.161 | -0.244  (-0.469, -0.019)  *p*=0.033 | -0.126  (-0.248, -0.004)  *p*=0.043 |
| *G4_3: Inclusion in Decision Making:* How included do you feel in the decision making of your community?^a^ | -1.119  (-1.372, -0.866)  *p*<0.0001 | -0.906  (-1.102, -0.709)  *p*<0.0001 | -0.929  (-1.200, -0.658)  *p*<0.0001 | -0.715  (-0.946, -0.485)  *p*<0.0001 | -0.993  (-1.296, -0.690)  *p*<0.0001 | -0.223  (-0.389, -0.058)  *p*=0.008 |

**^a^** Coded on a 5 point scale**:** 1**=**Not included at all; 2=A bit not included; 3=Neither included nor not included; 4=A bit included; 5=Very included

^b^ 1=Yes; 0=No

^c^ 1=Many (Yes, many/enough); 0=Not many (Yes, a few/not enough or No)

^d^ Average score of the two items; coded on a 4 point scale: 1=Never; 2=Not often; 3=Most of the time; 4=All the time

^e^ Coded on a 5 point scale**:** 1**=**Not at all; 2=Not very much; 3=No opinion; 4=A bit; 5=Completely

Given multiple comparisons *p*-values above 0.001 are not considered significant

1. We also assessed three additional Washington Group questions. The first asked about strength and identified eight additional cases of disability, all but one in the disabled group. Another asked how often respondents had fits or jerking body movements and identified thirty additional cases, 10 of these in the disabled groups. The final question asked respondents how often they felt worried, nervous or anxious which identified an additional 446 cases, the majority within the non-disabled group. Consequently, use of the full set of Washington Group questions identify 31% (*N* = 480) of respondents in control households as disabled. However, as this is twice as high as the current global estimate of 15%, and largely driven by one question on psychological wellbeing, we believe this estimate is likely over-sensitive in the present cultural context. [↑](#footnote-ref-1)
